# Urate, Blood Pressure, and Cardiovascular Disease: Evidence From Mendelian Randomization and Meta-Analysis of Clinical Trials

**DOI:** 10.1161/HYPERTENSIONAHA.120.16547

**Published:** 2020-12-28

**Authors:** Dipender Gill, Alan C. Cameron, Stephen Burgess, Xue Li, Daniel J. Doherty, Ville Karhunen, Azmil H. Abdul-Rahim, Martin Taylor-Rowan, Verena Zuber, Philip S. Tsao, Derek Klarin, VA Million Veteran Program, Evangelos Evangelou, Paul Elliott, Scott M. Damrauer, Terence J. Quinn, Abbas Dehghan, Evropi Theodoratou, Jesse Dawson, Ioanna Tzoulaki

**Affiliations:** 1 Department of Epidemiology and Biostatistics, School of Public Health (D.G., V.K., V.Z., E.E., P.E., A.D., I.T.), Imperial College London, United Kingdom.; 2 Department of Medicine, Centre for Pharmacology and Therapeutics, Hammersmith Campus (D.G.), Imperial College London, United Kingdom.; 3 MRC Centre for Environment and Health, School of Public Health (P.E., A.D., I.T.), Imperial College London, United Kingdom.; 4 British Heart Foundation Centre of Research Excellence (P.E.), Imperial College London, United Kingdom.; 5 Novo Nordisk Research Centre Oxford, Old Road Campus, United Kingdom (D.G.).; 6 Clinical Pharmacology and Therapeutics Section, Institute of Medical and Biomedical Education and Institute for Infection and Immunity, St George’s, University of London, United Kingdom (D.G.).; 7 Clinical Pharmacology Group, Pharmacy and Medicines Directorate, St George’s University Hospitals NHS Foundation Trust, London, United Kingdom (D.G.).; 8 Institute of Cardiovascular and Medical Sciences (A.C.C., D.J.D., M.T.-R., T.J.Q., J.D.), University of Glasgow, United Kingdom.; 9 Institute of Neuroscience and Psychology (A.H.A.-R.), University of Glasgow, United Kingdom.; 10 MRC Biostatistics Unit, Cambridge Institute of Public Health, United Kingdom (S.B., V.Z.).; 11 Cardiovascular Epidemiology Unit, Department of Public Health and Primary Care, University of Cambridge, United Kingdom (S.B.).; 12 Centre for Global Health, Usher Institute (X.L., E.T.), University of Edinburgh, United Kingdom.; 13 Edinburgh Cancer Research Centre, Institute of Genetics and Molecular Medicine (E.T.), University of Edinburgh, United Kingdom.; 14 School of Public Health, Zhejiang University, Hangzhou, China (X.L.).; 15 VA Palo Alto Health Care System, CA (P.S.T.).; 16 Department of Medicine, Stanford University School of Medicine, CA (P.S.T.).; 17 Malcom Randall VA Medical Center, Gainesville, FL (D.K.).; 18 Center for Genomic Medicine, Massachusetts General Hospital, Harvard Medical School, MA (D.K.).; 19 Program in Medical and Population Genetics, Broad Institute of MIT and Harvard, MA (D.K.).; 20 Division of Vascular Surgery and Endovascular Therapy, University of Florida School of Medicine, Gainesville, FL (D.K.).; 21 Department of Hygiene and Epidemiology, University of Ioannina Medical School, Greece (E.E., I.T.).; 22 UK Dementia Research Institute at Imperial College London, United Kingdom (P.E., A.D., I.T.).; 23 Imperial Biomedical Research Centre, Imperial College London and Imperial College NHS Healthcare Trust, United Kingdom (P.E., I.T.).; 24 Health Data Research UK, London (P.E.).; 25 Corporal Michael J. Crescenz VA Medical Center, PA (S.M.D.).; 26 Department of Surgery, University of Pennsylvania Perelman School of Medicine, Philadelphia (S.M.D.).

**Keywords:** blood pressure, cardiovascular diseases, odds ratio, systematic review, uric acid

## Abstract

Serum urate has been implicated in hypertension and cardiovascular disease, but it is not known whether it is exerting a causal effect. To investigate this, we performed Mendelian randomization analysis using data from UK Biobank, Million Veterans Program and genome-wide association study consortia, and meta-analysis of randomized controlled trials. The main Mendelian randomization analyses showed that every 1-SD increase in genetically predicted serum urate was associated with an increased risk of coronary heart disease (odds ratio, 1.19 [95% CI, 1.10–1.30]; *P*=4×10^−5^), peripheral artery disease (1.12 [95% CI, 1.03–1.21]; *P*=9×10^−3^), and stroke (1.11 [95% CI, 1.05–1.18]; *P*=2×10^−4^). In Mendelian randomization mediation analyses, elevated blood pressure was estimated to mediate approximately one-third of the effect of urate on cardiovascular disease risk. Systematic review and meta-analysis of randomized controlled trials showed a favorable effect of urate-lowering treatment on systolic blood pressure (mean difference, −2.55 mm Hg [95% CI, −4.06 to −1.05]; *P*=1×10^−3^) and major adverse cardiovascular events in those with previous cardiovascular disease (odds ratio, 0.40 [95% CI, 0.22–0.73]; *P*=3×10^−3^) but no significant effect on major adverse cardiovascular events in all individuals (odds ratio, 0.67 [95% CI, 0.44–1.03]; *P*=0.07). In summary, these Mendelian randomization and clinical trial data support an effect of higher serum urate on increasing blood pressure, which may mediate a consequent effect on cardiovascular disease risk. High-quality trials are necessary to provide definitive evidence on the specific clinical contexts where urate lowering may be of cardiovascular benefit.

## Abstract 2

Supplemental Digital Content is available in the text.

Urate is a breakdown product of purine metabolism. Its raised levels have been associated with a number of adverse health outcomes including gout, hypertension, and cardiovascular disease (CVD).^1^ However, it remains unclear whether these associations represent causal effects.^2,3^ The relationship between serum urate, obesity, diet, and other cardiovascular risk factors raises considerable potential for confounding and reverse causation.^4^

The genetic determinants of serum urate levels have been increasingly well-characterized.^4^ This has made it possible to identify better instruments for Mendelian randomization (MR) analyses investigating the effect of genetically predicted serum urate on cardiovascular outcomes than in previous efforts.^3,5–7^ The use of variants randomly allocated at conception to proxy the effect of modifying serum urate means that MR is less susceptible to the environmental confounding, measurement error, and reverse causation bias that can limit causal inference in traditional epidemiological approaches.

Preclinical studies support a causal role for urate in hypertension.^8^ Randomized controlled trial (RCT) data have shown that both allopurinol and probenecid reduce systolic blood pressure (SBP) in hyperuricemic adolescents.^1,9,10^ Pooling of all available trial data can offer more precise effect estimates for the effect of urate-lowering therapy on blood pressure.^11^ As elevated blood pressure is a risk factor for CVD, it is important to clarify any role of SBP in mediating an effect of urate on cardiovascular outcomes.

The aim of the current study was to perform MR analyses investigating the association of genetically predicted urate levels with SBP and CVD risk using contemporary data and compare findings with results obtained from updated systematic review and meta-analysis of urate-lowering RCTs.

## Methods

All data used in this work are either publicly accessible or available on request from their original studies, which obtained appropriate patient consent and ethical approval. The UK Biobank data were accessed through application 236. All results generated in this work are presented in either the article or its Data Supplement.

### Two-Sample MR

The MR analyses have been reported as per the Strengthening the Reporting of Observational Studies in Epidemiology-Mendelian randomization guidelines.^12^

### Genetic Association Estimates

Genetic association estimates for serum urate in 2-sample MR were obtained by using the PLINK software to meta-analyze summary data from genome-wide association study (GWAS) analyses of 110 347 European-ancestry individuals and 343 836 White British UK Biobank participants, respectively.^13–15^ Urate estimates in MR analyses are provided per 1-SD increase, which corresponds to 80.3 μmol/L. For consideration of SBP (or diastolic blood pressure/pulse pressure) as a mediator in the 2-sample multivariable MR, genetic association estimates were obtained from a GWAS of 317 195 White British UK Biobank participants, where blood pressure was measured using automated readings with correction made for any antihypertensive drug use by adding 10 mm Hg to the measured reading for SBP and 5 mm Hg for diastolic blood pressure.^16^ In contrast, when investigating SBP as an outcome, genetic association estimates were obtained from the International Consortium for Blood Pressure GWAS analysis of 287 245 European-ancestry individuals (excluding UK Biobank participants).^17^ A different population was considered when studying SBP as an outcome to avoid overlap with the UK Biobank participants used to obtain genetic association estimates for urate, as this can bias MR estimates.^18^ SBP estimates are provided per 1-SD increase, which corresponds to 18.6 mm Hg. Genetic association estimates for CHD were obtained from the CARDIoGRAMplusC4D Consortium 1000G multiethnic GWAS (77% European ancestry) of 60 801 cases and 123 504 controls, with a broad and inclusive definition of CHD applied.^19^ Genetic association estimates for peripheral artery disease (PAD) were obtained from the Million Veterans Program multiethnic (72% European ancestry) GWAS of 31 307 cases and 211 753 controls, with case definitions made using hospital diagnosis and procedure codes.^20^ Genetic association estimates for stroke were obtained from the MEGASTROKE multiethnic (86% European ancestry) GWAS of 67 162 cases and 454 450 controls,^21^ with the stroke definition including both ischemic and hemorrhagic etiologies. Genetic association estimates for ischemic stroke only (60 341 cases and 454 450 controls) were used in secondary analyses.^21^

### Genetic Variants Used as Instruments

Genetic instruments for the 2-sample MR were identified as single-nucleotide polymorphisms that were associated with urate (or SBP, in mediation analyses) at genome-wide significance (*P*<5×10^−8^) and were in pair-wise linkage disequilibrium with *r*^2^<0.001. Clumping was performed using the TwoSampleMR package of R.^22^ For univariable MR, instrument strength was estimated using the F statistic, with variance in the exposure explained assessed using the *R*^2^ value.^23^

### Statistical Analysis

In all analyses, single-nucleotide polymorphisms were aligned by their effect alleles, and no additional consideration was made for palindromic variants. Two-sample MR analyses were performed to investigate the effect of genetically predicted serum urate on CHD, PAD, stroke, and SBP, respectively. A Bonferroni threshold (*P*<0.01) that corrected for multiple testing related to the 4 outcomes was used to ascertain statistical significance in the main analysis. Inverse-variance weighted (IVW) MR was used in the main analysis, with the simple median, contamination mixture method, Egger, pleiotropy residual sum and outlier,^24^ and multivariable MR^25^ (only for CVD outcomes) sensitivity analyses used to explore the robustness of the findings to potential pleiotropy of the genetic variants. Given the previously demonstrated overlap in the genetic determinants of urate with other metabolic traits,^4^ the multivariable MR sensitivity analysis adjusted for genetic associations of the instruments with body mass index, estimated glomerular filtration rate, type 2 diabetes, serum low-density lipoprotein cholesterol, serum high-density lipoprotein cholesterol, and serum triglycerides together in the same model. Such multivariable MR was not performed when considering SBP as an outcome, due to population overlap with the cohorts used to obtain genetic association estimates for the metabolic exposures.^18^ In MR mediation analyses, multivariable MR was applied in the 2-sample setting to adjust for the genetic association of the instruments with SBP (or diastolic blood pressure/pulse pressure). Network MR was used to estimate the proportion of the total effect of urate on each cardiovascular outcome that is mediated through SBP.^26^ SEs were estimated using the propagation of error method.

To investigate potential effects of the cardiovascular traits on serum urate levels, we also performed IVW MR analyses considering genetically predicted CHD, PAD, stroke, and SBP as exposures and serum urate levels as the outcome. The approach taken was as described above, except that the considered exposure and outcome were switched.

Further details on the 2-sample MR analyses are provided in Methods in the Data Supplement. Statistical analyses for the 1-sample MR are detailed in Methods in the Data Supplement and Table S1 in the Data Supplement.

### Systematic Review and Meta-Analysis of RCTs

The systematic review was conducted in accordance with the recommendations from the Preferred Reporting Items for Systematic Reviews and Meta-Analyses statement.^27^ The study protocol was registered and published in the International Prospective Register of Systematic Reviews (CRD42020164589).

### Inclusion and Exclusion Criteria

RCTs of pharmacological urate-lowering versus placebo or no treatment of duration ≥28 days in patients ≥18 years of age were eligible. Studies with cointervention that was inconsistent between intervention and control groups or with mixed experimental groups were excluded.

### Data Sources and Searches

We conducted a systematic search of multidisciplinary databases across 4 electronic platforms: Medline (Ovid), Embase (Ovid), Web of Science (Thomson Reuters), and Cochrane Library (Cochrane). Studies published from January 1, 2016, to September 30, 2019, were considered. The literature search terms matched those used in a previous systematic review (Methods in the Data Supplement).^10^ No language restrictions were applied. Reference lists of retrieved articles were reviewed to identify additional relevant articles. Data were included from studies in previous systematic reviews investigating the effects of urate-lowering therapy on SBP (searches until June 29, 2016)^11^ and CVD (searches until December 30, 2016).^7^

### Data Extraction

Study details and results were extracted using a predesigned template. In the case of crossover trials, only data from the first study period (before crossover) were used. Risk of bias was assessed in accordance with the Cochrane guidelines.^28^ Studies were considered to have a low overall risk of bias if the risk of bias was low for all key study domains. All aspects of the database search, study selection, data extraction, and risk of bias assessment were performed independently by 2 investigators (A.C.C. and D.J.D.), with arbitration to a third investigator (A.H.A.-R.) as necessary.

### Outcomes

The outcomes considered in the systematic review and meta-analysis of RCTs were SBP and risk of major adverse cardiovascular event (MACE; cardiovascular death, nonfatal myocardial infarction, unstable angina requiring urgent revascularization, or nonfatal stroke). In secondary analysis, we also considered MACE in patients with prior CVD, as this would likely represent a higher risk population, thus potentially offering greater statistical power.

### Statistical Analysis

Analysis of extracted data was based on modified intention-to-treat (considering patients who received at least 1 dose of the allocated treatment) or intention-to-treat results. If not reported, the mean change in SBP pre- and post-treatment was calculated and the SD was imputed using the propagation of error method using a correlation coefficient that was derived from the largest study in the meta-analysis that reported SD for SBP values at baseline, at end of the study, and for the change in SBP.^29,30^ The measure of effect size was the difference in mean SBP, defined as the mean difference between patients treated with urate-lowering therapy versus control. A negative value of the difference in mean SBP indicates a greater reduction of SBP in the urate-lowering therapy group as compared with the control group. Overall CVD risk estimates are described in terms of odds ratio (OR).

Random-effects IVW meta-analysis was used to pool estimates from different studies, and study heterogeneity was assessed using the I^2^ statistic. Cochran Q test was used to investigate heterogeneity between the meta-analysis estimates obtained for risk of MACE in all patients as compared with those with previous CVD. Sensitivity analyses were conducted including only studies at low risk of bias. For the analysis considering SBP as an outcome, meta-regression was performed to assess the effect of baseline SBP on treatment response. Meta-regression analyses were also performed to assess the associations of baseline serum urate concentration, absolute change in serum urate concentration, and proportional change in serum urate concentration with treatment response for changes in SBP and risk of MACE in all patients. Analyses were conducted using the Comprehensive Meta-Analysis software, version 3 (Biostat).

## Results

### Mendelian Randomization

**Figure 1.**
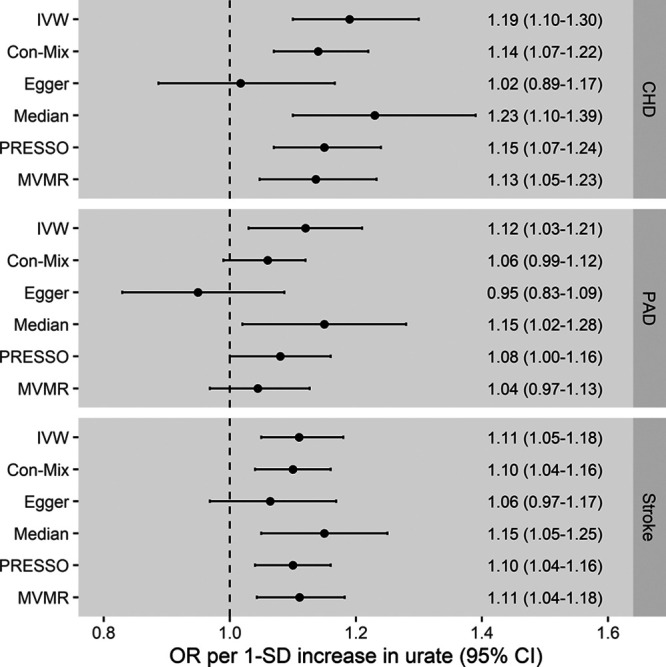
**Mendelian randomization estimates for the effect of 1-SD increase in genetically predicted serum urate levels on risk of coronary heart disease (CHD), peripheral artery disease (PAD), and stroke.** Multivariable Mendelian randomization (MVMR) adjusting for genetic associations of the instrument variants with body mass index, estimated glomerular filtration rate, type 2 diabetes, low-density lipoprotein cholesterol, high-density lipoprotein cholesterol, and triglycerides. The outlier-corrected pleiotropy residual sum and outlier (PRESSO) results are presented (5 outlier variants were identified for CHD, 8 for PAD, and 1 for stroke). The Egger intercept was 0.005 ([95% CI, 0.001–0.009] *P*=0.004) for CHD, 0.005 ([95% CI, 0.001–0.009] *P*=0.003) for PAD, and 0.001 ([95% CI, −0.001 to 0.003] *P*=0.24) for stroke. Con-Mix indicates contamination mixture; IVW, inverse-variance weighted; and OR, odds ratio.

**Figure 2.**
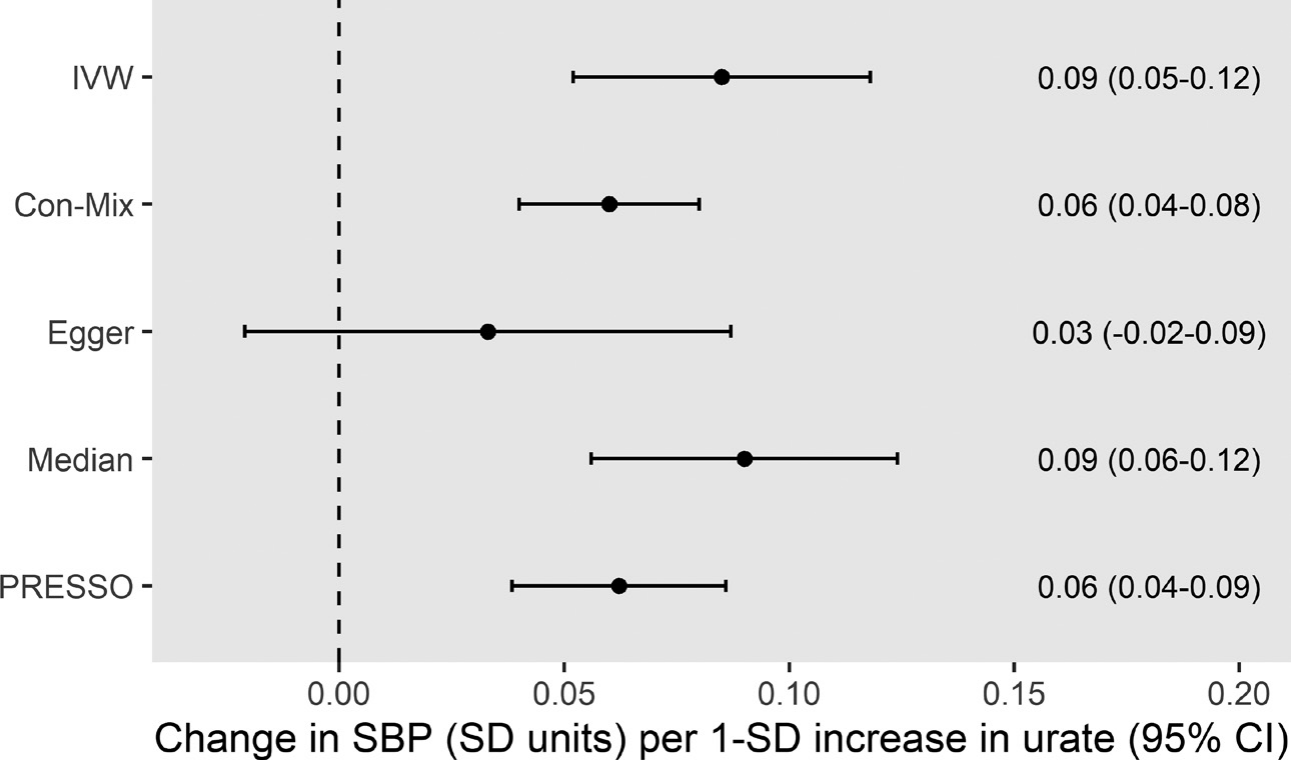
**Mendelian randomization estimates for the effect of 1-SD increase in genetically predicted serum urate levels on systolic blood pressure.** The outlier-corrected pleiotropy residual sum and outlier (PRESSO) results are presented (21 outlier variants were identified). The Egger intercept was 0.002 ([95% CI, 0.0004–0.003] *P*=0.02). Con-Mix indicates contamination mixture; IVW, inverse-variance weighted; and SBP, systolic blood pressure.

The instruments for urate that were used in the 2-sample MR are presented in Table S2. The main IVW MR showed that higher genetically predicted serum urate levels were associated with an increased risk of CHD, with OR of 1.19 ([95% CI, 1.10–1.30] *P*=4×10^−5^) per 1-SD increase in genetically predicted urate. Consistent results were obtained in all MR sensitivity analyses except MR-Egger, which had wide 95% CIs (Figure 1). Higher genetically predicted serum urate was also associated with increased risk of both PAD (OR, 1.12 [95% CI, 1.03–1.21]; *P*=9×10^−3^) and stroke (OR, 1.11 [95% CI, 1.05–1.18]; *P*=2×10^−4^) in the main IVW analysis, with similar results obtained in all MR sensitivity analyses except MR-Egger, which again had wide 95% CIs (Figure 1). For the multivariable MR adjusting for genetic confounding through body mass index, estimated glomerular filtration rate, type 2 diabetes, and lipid traits, direct effects of these exposures on risk of the respective CVD outcomes are presented in Table S3. Considering SBP, the main IVW and sensitivity MR analyses all provided supporting evidence of a causal effect of serum urate (IVW estimate in SD units per 1-SD increase in genetically predicted urate, 0.09 [95% CI, 0.05–0.12]; *P*=6×10^−^^7^; Figure 2). Scatter plots depicting the association of the instrument variants with serum urate and the respective outcomes are presented in Figures S1 through S4.

**Figure 3.**
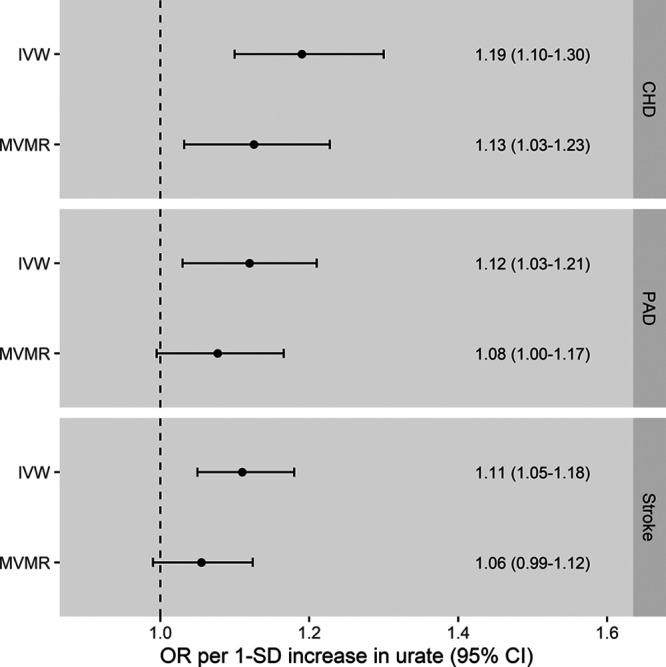
**Inverse-variance weighted (IVW) and multivariable Mendelian randomization (MVMR) estimates for the effect of 1-SD increase in genetically predicted serum urate levels on risk of coronary heart disease (CHD), peripheral artery disease (PAD), and stroke.** The MVMR analysis adjusts for the associations of the genetic instruments with systolic blood pressure. The MVMR only adjusted for the associations of the genetic instruments with systolic blood pressure and not any other trait. OR indicates odds ratio.

Performing multivariable MR to adjust for genetically predicted SBP showed attenuation of the urate effect estimates for the CVD outcomes as compared with the main IVW univariable MR (Figure 3), supporting that part of the effect of urate on these outcomes is mediated through SBP. Similar results were obtained when considering diastolic or pulse pressure as mediators (Figure S5) or when considering only ischemic stroke rather than all stroke as an outcome (Figure S6). Network MR mediation analysis quantified this as 29% (95% CI, 9%–48%) for CHD, 44% (95% CI, 5%–83%) for PAD, and 45% (95% CI, 14%–76%) for stroke. For CHD, there remained evidence of a direct effect of urate even after adjusting for SBP (OR, 1.13 [95% CI, 1.03–1.23]; *P*=0.01). In contrast for PAD and stroke, although the estimate for the direct effect of urate that is not mediated through SBP was positive, the CI crossed the null and the results were not statistically significant (PAD: OR, 1.08 [95% CI, 1.00–1.17]; *P*=0.07; stroke: OR, 1.06 [95% CI, 0.99–1.12]; *P*=0.10). Direct effects of SBP on the outcomes after adjusting for genetically predicted serum urate are presented in Table S4.

There was no evidence of associations between genetically proxied CHD, PAD, stroke, or SBP with serum urate levels (Table S5). The results of the 1-sample MR analysis were consistent with the abovementioned 2-sample MR analysis and are detailed in Results in the Data Supplement and Tables S6 through S8. Results were similar for men and women in sex-stratified analyses (Results in the Data Supplement).

### Systematic Review and Meta-Analysis of RCTs

In total, 92 studies were eligible for the systematic review and meta-analysis. Regarding the updated searches (January 1, 2016, to September 30, 2019), 5353 records were screened, 30 full texts were assessed for eligibility, and 13 studies were identified that fulfilled the eligibility criteria (Figure S7). Characteristics of included studies that were identified in the updated search are presented in Table S9.

**Figure 4.**
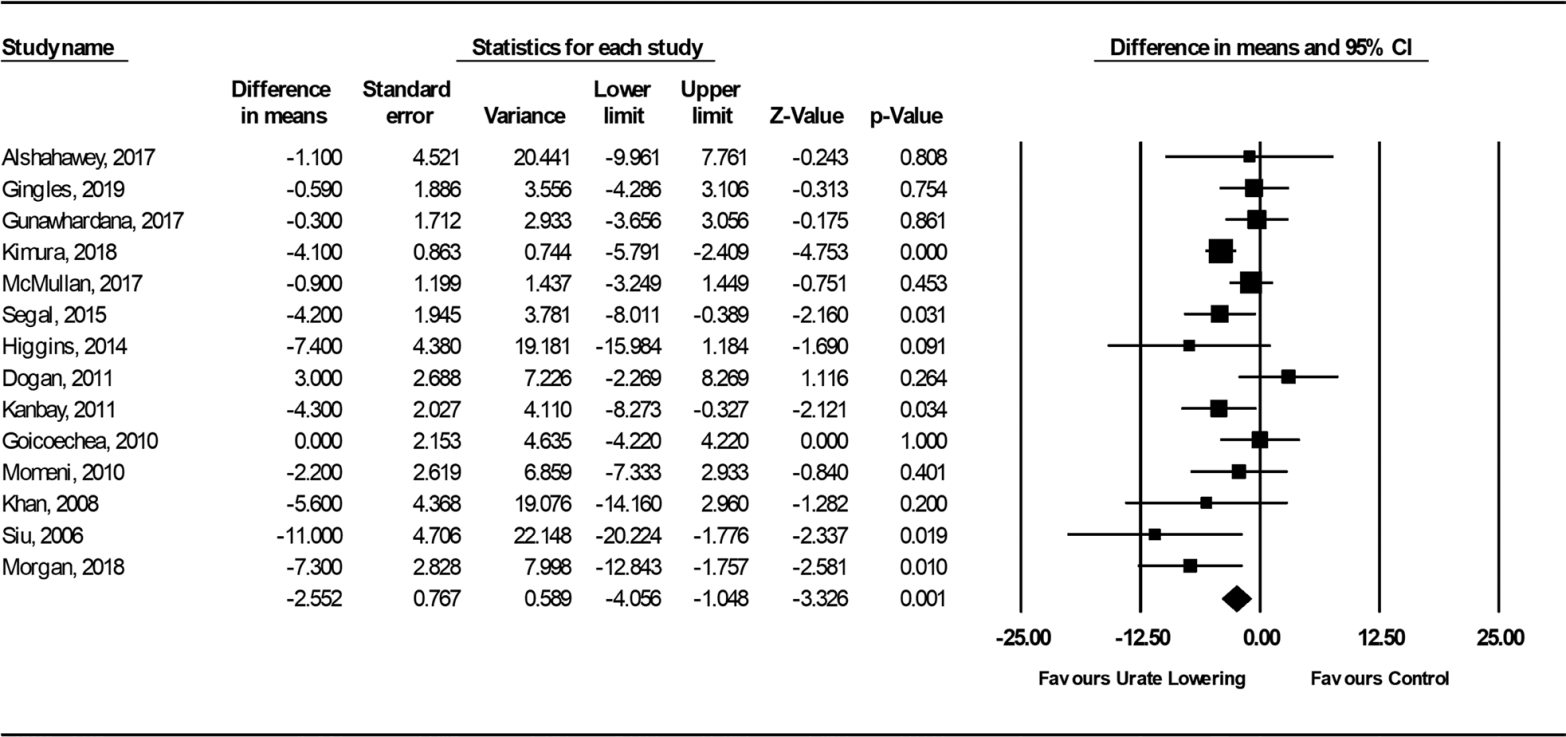
**Forest plot of randomized controlled trial estimates for change in mean systolic blood pressure in patients receiving urate-lowering therapy or placebo/no treatment.** I^2^ heterogeneity statistic: 43%. A random-effects meta-analysis model was used.

**Figure 5.**
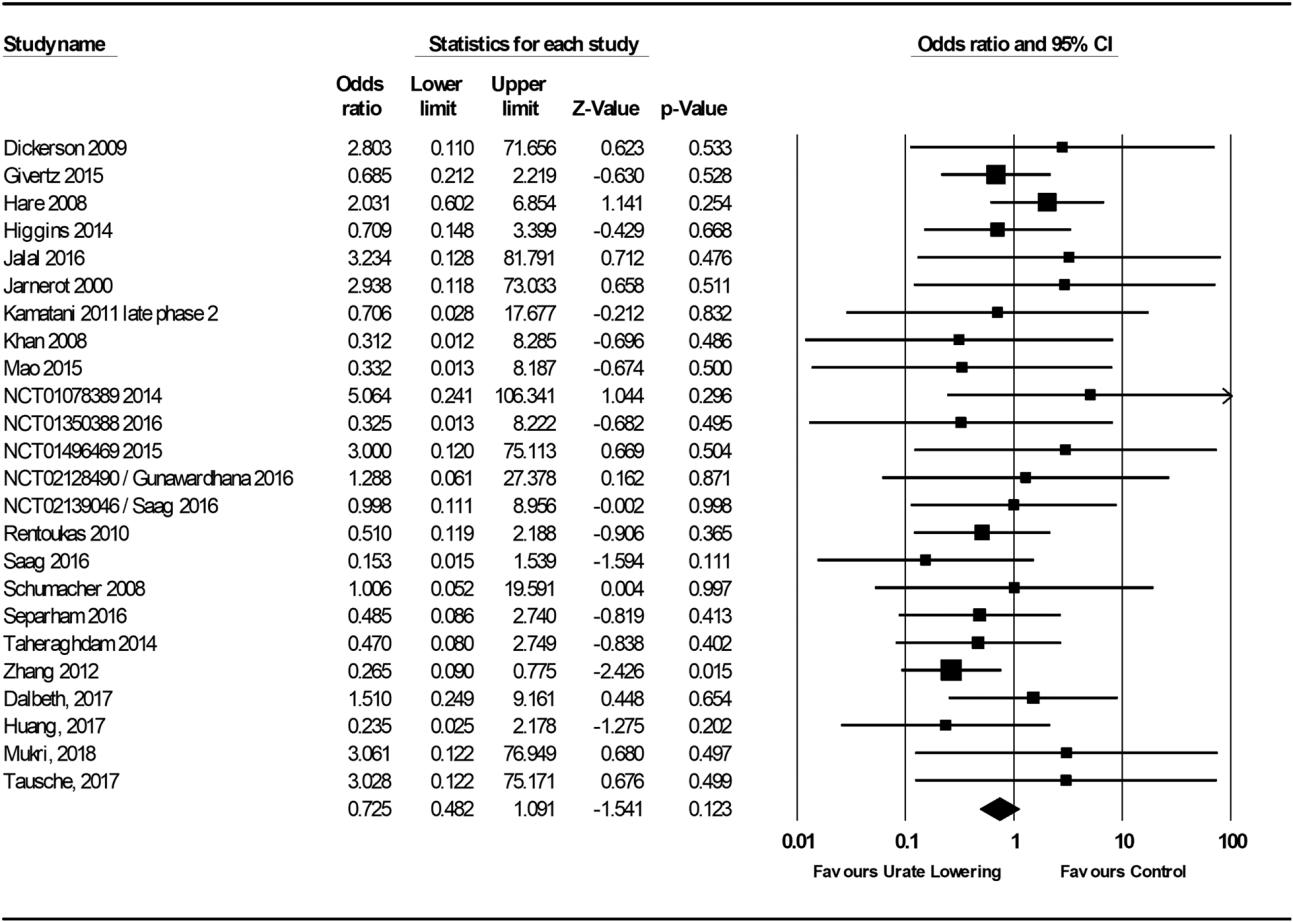
**Forest plot of randomized controlled trial estimates for risk of major adverse cardiovascular events in all patients receiving urate-lowering therapy or placebo/no treatment.** I^2^ heterogeneity statistic: 0%. A random-effects meta-analysis model was used.

**Figure 6.**
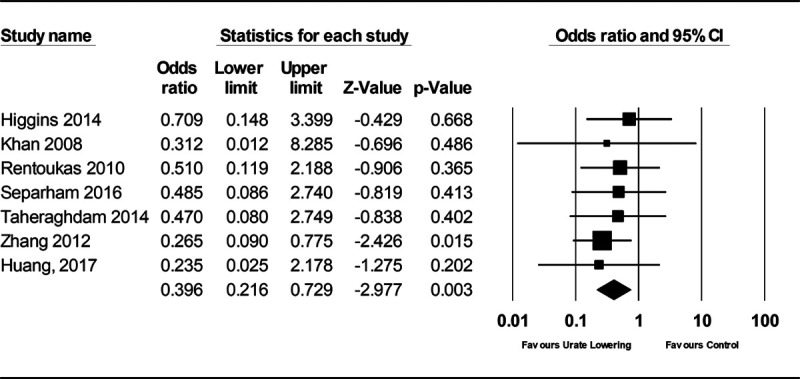
**Forest plot of randomized controlled trial estimates for risk of major adverse cardiovascular events in patients with existing cardiovascular disease receiving urate-lowering therapy or placebo/no treatment.** I^2^ heterogeneity statistic: 0%. A random-effects meta-analysis model was used.

The primary analysis of effects on SBP included 15 studies (Figure S8). The SD for mean SBP change pre- and post-treatment was only available for 1 study,^29^ and this was used to impute the SD for mean SBP change pre- and post-treatment for the other studies.^30^ There was heterogeneity between the 15 studies included (I^2^=89%), which was mostly attributable to 1 study considering renal dialysis patients that hid a high risk of bias (Table S10; Figure S8).^31^ Excluding this study, subjects treated with urate-lowering therapy had greater reduction in SBP than subjects in the control group (mean difference in SBP, −2.55 [95% CI, −4.06 to −1.05]; *P*=1×10^−^^3^; I^2^=43%; Figure 4). The analysis of MACE risk in all patients considered 85 studies, of which 21 had events (Figure 5), and the analysis of MACE risk in patients with prior cardiovascular events considered 10 studies, of which 7 had events (Figure 6). Urate-lowering therapy was not significantly associated with risk of MACE in all patients (OR, 0.67 [95% CI, 0.44–1.03]; *P*=0.07; I^2^=0%) but was significantly associated with reduction in risk of MACE in patients with a prior cardiovascular event (OR, 0.40 [95% CI, 0.22–0.73]; *P*=3×10^−3^; I^2^=0%). There was no evidence of heterogeneity between the estimates obtained from studies considering all patients as compared with those restricted to patients with prior CVD (Cochran Q *P*=0.11).

Risk of bias was low in 16 of the 92 included studies (Table S10). Risk of bias was low in 1 of the 15 studies investigating effects on SBP, 16 of the 24 studies investigating MACE in all patients, and 2 of the 7 studies investigating MACE in patients with a prior cardiovascular event. In sensitivity analyses including only studies at low-risk bias, urate-lowering therapy was not significantly associated with greater SBP lowering compared with control (mean difference in SBP, −7.40 [95% CI, −15.98 to 1.18]; *P*=0.09), MACE in all patients (OR, 0.64 [95% CI, 0.28–1.46]; *P*=0.29; I^2^=0%; Figure S9), or MACE in patients with a prior cardiovascular event (OR, 0.59 [95% CI, 0.18–1.91]; *P*=0.38; I^2^=0%; Figure S10).

Meta-regression analysis demonstrated no association of baseline SBP with SBP change following urate-lowering therapy (coefficient, −0.01 [95% CI, −0.19 to 0.17]; *P*=0.92; Figure S11). The serum urate concentration entry criteria for inclusion in the studies, mean baseline serum urate concentration, and absolute and proportional change in serum urate concentration from baseline for studies included in the systematic review are presented in Table S11. Meta-regression demonstrated that higher baseline serum urate concentration was associated with greater SBP reduction with urate-lowering therapy (coefficient, −1.70 [95% CI, −2.81 to −0.60]; *P*=2×10^−3^; Figure S12). Meta-regression did not identify an association of absolute change in serum urate concentration (*P*=0.63) or proportional change in serum urate concentration (*P*=0.59) with SBP change with urate-lowering therapy. Meta-regression did not identify an association of baseline serum urate concentration (*P*=0.96), absolute change in urate concentration (*P*=0.71), or proportional change in serum urate concentration (*P*=0.45) with change in MACE risk with treatment with urate-lowering therapy.

## Discussion

We used a comprehensive framework of MR methodologies to perform detailed investigation into the association of genetically predicted serum urate with CVD outcomes and replicated findings in the independent UK Biobank population. The MR analyses went beyond recent efforts, to consider genetic association estimates from larger studies of serum urate. The MR analyses identified consistent evidence for an association between higher genetically predicted serum urate levels with risk of CVD and SBP. Performing multivariable MR to adjust for genetic association with SBP attenuated the estimates for the CVD outcomes to support that at least some of the effect of urate may be mediated through raised blood pressure. Updated systematic review and meta-analysis of RCT data similarly showed a favorable effect of urate-lowering treatment on SBP, with meta-regression supporting a greater effect in individuals with higher baseline urate. There was also some trial evidence to support a protective effect on MACE risk in individuals with prior CVD.

Hyperuricemia has been postulated to cause endothelial dysfunction by increasing oxidative stress,^1^ and this could directly increase risk of CVD through effects on the vascular endothelium.^1^ In an animal model where hyperuricemia was induced using a uricase inhibitor, hypertension followed after 3 weeks while controls remained normotensive,^8^ thus representing a second mechanism by which urate can increase risk of CVD. The results of the FAST (Febuxostat and Allopurinol Streamlined Trial) and HEART trials (Allopurinol and Cardiovascular Outcomes in Patients With Ischaemic Heart Disease) will help provide further insight on potential repurposing of existing urate-lowering agents for CVD prevention.^32,33^ Allopurinol could represent an inexpensive, safe, and well-tolerated drug for reducing cardiovascular risk.^34^

Our current study has several strengths. We triangulate evidence across MR analyses and RCT data to provide consistent evidence for a role of serum urate in increasing SBP and potentially also increasing CVD risk. Our analyses identify a possible mediating role of SBP in the pathway from serum urate to CVD risk. In contrast, previous MR studies have considered associations of the genetic instruments for urate with blood pressure as representing genetic confounding.^3,5–7^ Exclusion of such variants or adjustment for their genetic association with blood pressure traits, as was done in these studies,^3,5–7^ would obscure any true causal effect of genetically predicted urate on CVD risk. Our present MR analysis also incorporated more powerful instruments for serum urate than were available previously. The variants used as instruments in our 1-sample MR analysis of UK Biobank participants explained ≈7.7% of the variance in serum urate,^4^ in contrast to the 5.3% that would be explained if selecting instrument variants from the previous largest published GWAS of serum urate.^13^ To further advance on previous MR efforts,^3,6^ our study also incorporated formal mediation analyses to investigate blood pressure as a mediator in the effect of serum urate on CVD risk. Similarly, our systematic review and meta-analysis of RCTs updated evidence from previous efforts to consider an additional 13 studies.^10,11^

### Study Limitations

Our work also has limitations. The MR approach makes a series of modeling assumptions and in particular requires that the genetic variants used as instruments do not affect the considered outcomes through pathways that are independent of urate. While this can never be completely excluded, we performed a range of MR sensitivity analyses that make distinct assumptions on the presence of pleiotropic variants and generally found consistent estimates. The MR-Egger method generated wide 95% CIs in all analyses and was likely of limited reliability due to the strength of the urate instruments being correlated to their direct effects on the CVD outcomes under consideration.^4,35^ The genetic analyses were also based predominantly on individuals of European ancestry and thus may not apply to other populations or settings. Finally, use of urate-lowering mediation was not accounted for in MR analyses. Considering the meta-analysis of RCTs, the majority (76 of 92) of studies had a high or undetermined risk of bias, and there was heterogeneity in the results of RCTs measuring change in SBP following urate-lowering therapy, possibly related to study design, in turn limiting the strength of conclusions that can be drawn. Renal dialysis patients were not included in the MR analyses, and these results may, therefore, not apply to such populations. Finally, it may be that the identified effect of urate lowering on MACE in meta-analysis of trials considering patients with previous CVD but not in all patients or when restricting to studies with low risk of bias was attributable to insufficient statistical power.

### Perspectives

We have found consistent MR and RCT evidence for an effect of higher serum urate levels on increasing SBP, with further evidence also supporting a potential effect on risk of CVD. High-quality trial data are now necessary to provide definitive evidence on the specific clinical contexts where urate lowering may be of cardiovascular benefit, with some large-scale studies already underway.^32,33^

## Acknowledgments

We acknowledge the contributors of the data used in this work: CARDIoGRAMplusC4D, CKDGen, DIAGRAM, Genetic Investigation of Anthropometric Traits, Global Lipids Genetics Consortium, International Consortium for Blood Pressure, MEGASTROKE, Million Veterans Program, and UK Biobank. D. Gill, J. Dawson, A.C. Cameron, E. Theodoratou, and I. Tzoulaki designed the study. D. Gill, X. Li, A.C. Cameron, D.J. Doherty, A.H. Abdul-Rahim, and M. Taylor-Rowan had full access to the data and performed the analysis. All authors interpreted the results. D. Gill, J. Dawson, A.C. Cameron, and I. Tzoulaki drafted the manuscript. All authors critically revised the manuscript for intellectual content. All authors approved the submitted version and are accountable for the integrity of the work.

## Sources of Funding

This work was supported by funding from the US Department of Veterans Affairs Office of Research and Development, Million Veteran Program Grant MVP003 (I01-BX003362), and the UK National Institute for Health Research Cambridge Biomedical Research Centre. This publication does not represent the views of the Department of Veterans Affairs of the US Government. D. Gill is funded by the Wellcome 4i Clinical PhD Program (203928/Z/16/Z) and the British Heart Foundation Centre of Research Excellence (RE/18/4/34215) at Imperial College London. S. Burgess is supported by Sir Henry Dale Fellowship jointly funded by the Wellcome Trust and the Royal Society (grant No. 204623/Z/16/Z). V. Karhunen is funded by the European Union Horizon 2020 research and innovation program under the Marie Sklodowska-Curie grant (721567). P. Elliott acknowledges support from the British Heart Foundation (RE/18/4/34215), the Medical Research Council (MR/S019669/1), the National Institute for Health Research Imperial Biomedical Research Centre, Imperial College London (RDF03), the UK Dementia Research Institute (DRI) at Imperial College London funded by UK DRI, Ltd (funded by the Medical Research Council, Alzheimer’s Society, Alzheimer’s Research UK), and Health Data Research (HDR) UK London funded by HDR UK, Ltd (funded by a consortium led by the Medical Research Council 1004231). S.M. Damrauer was supported by the Department of Veterans Affairs Office of Research and Development (IK2-CX001780). E. Theodoratou is funded by a Cancer Research UK Career Development Fellowship (C31250/A22804). The MEGASTROKE project received funding from sources specified at http://www.megastroke.org/acknowledgments.html. Details of all MEGASTROKE authors are available at http://www.megastroke.org/authors.html.

## Disclosures

D. Gill is employed part-time by Novo Nordisk outside the submitted work. E. Evangelou acknowledges consultancy fees from OpenDNA outside of the submitted work. S.M. Damrauer has received grants from the US Department of Veterans Affairs and Renalytix AI plc outside the submitted work. J. Dawson and T.J. Quinn have received charitable research income to conduct clinical trials of allopurinol use in people with stroke. The other authors report no conflicts.

## Supplementary Material



## Novelty and Significance

### What Is New?

- This study found that higher genetically predicted serum urate was associated with an increased risk of cardiovascular disease, with elevated blood pressure mediating approximately one-third of this effect.
- Meta-analysis of randomized controlled trials showed a favorable effect of urate lowering on systolic blood pressure and cardiovascular events in those with previous cardiovascular disease but no significant effect on cardiovascular events in all individuals.

### What Is Relevant?

- Lowering serum urate may represent a therapeutic target for reducing blood pressure and risk of cardiovascular disease.

### Summary

Genetic analyses and clinical trial data support an effect of higher serum urate on increasing blood pressure, which may mediate a consequent effect on cardiovascular disease risk. High-quality trials are necessary to provide definitive evidence on the specific clinical contexts where urate lowering may be of cardiovascular benefit.
