# Supplementary figures and images for: Urate, Blood Pressure, and Cardiovascular Disease: Evidence From Mendelian Randomization and Meta-Analysis of Clinical Trials
Source: Hypertension. 2020 Dec 28;77(2):383–92. doi: 10.1161/HYPERTENSIONAHA.120.16547 (PMC7803439; doi:10.1161/HYPERTENSIONAHA.120.16547)

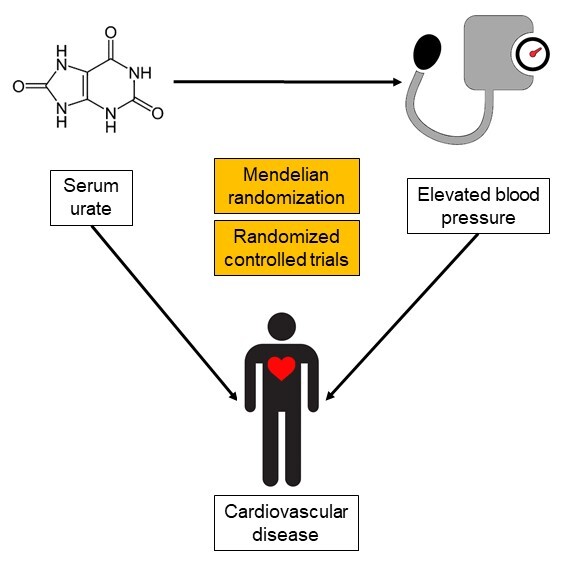

Supplement: Supplementary file 1 [file hyp-77-383-s001.jpg]
