# Supplementary material for: Urate, Blood Pressure, and Cardiovascular Disease: Evidence From Mendelian Randomization and Meta-Analysis of Clinical Trials
Source: Hypertension. 2020 Dec 28;77(2):383–92. doi: 10.1161/HYPERTENSIONAHA.120.16547 (PMC7803439; doi:10.1161/HYPERTENSIONAHA.120.16547)
Supplement: Supplementary file 2 [file hyp-77-383-s002.doc]

# **Supplement**

**Urate, blood pressure and cardiovascular disease: evidence from Mendelian randomization and meta-analysis clinical trials**

Gill *et al*. Urate, blood pressure and cardiovascular disease

### **Corresponding author**

Dr Dipender Gill

Department of Epidemiology and Biostatistics, School of Public Health, Medical School Building, St Mary's Hospital, Imperial College London, United Kingdom, W2 1PG

Telephone: +44 (0) 7904843810

Fax: +44 (0) 20 3313 8223

E-mail: [dipender.gill@imperial.ac.uk](mailto:dipender.gill@imperial.ac.uk)

**Contents**

[**Urate, blood pressure and cardiovascular disease: evidence from Mendelian randomization and meta-analysis clinical trials 1**](#__RefHeading___Toc57189268)

[**Corresponding author 1**](#__RefHeading___Toc57189269)

[**Supplementary Methods 4**](#__RefHeading___Toc57189270)

[**Two-sample Mendelian randomization analysis 4**](#__RefHeading___Toc57189271)

[**One-sample Mendelian randomization analysis 5**](#__RefHeading___Toc57189272)

[**Systematic review and meta-analysis of randomized controlled trials 7**](#__RefHeading___Toc57189273)

[**Supplementary Results 11**](#__RefHeading___Toc57189274)

[**One-sample Mendelian randomization 11**](#__RefHeading___Toc57189275)

[**References 12**](#__RefHeading___Toc57189276)

[**Table S1 – International Classification of Diseases version 10 (ICD-10) codes used to identify incident cardiovascular disease events 15**](#__RefHeading___Toc57189277)

[**Table S2 – Two-sample Mendelian randomization instruments for urate 16**](#__RefHeading___Toc57189278)

[**Table S3 – Multivariable Mendelian randomization estimates for the direct effect of serum urate and metabolic traits, on risk of coronary heart disease (CHD), peripheral arterial disease (PAD) and stroke. 22**](#__RefHeading___Toc57189279)

[**Table S4 – Multivariable Mendelian randomization estimates for the direct effect of 1-standard deviation increase in genetically determined serum urate and systolic blood pressure (SBP) respectively, on risk of coronary heart disease (CHD), peripheral arterial disease (PAD) and stroke. 23**](#__RefHeading___Toc57189280)

[**Table S5 – Inverse-variance weighted Mendelian randomization analyses investigating the association of genetically proxied coronary heart disease (CHD), peripheral arterial disease (PAD), stroke and systolic blood pressure (SBP) on serum urate levels. 24**](#__RefHeading___Toc57189281)

[**Table S6 – Characteristics of the UK Biobank populations considered and analyzed in one-sample Mendelian randomization. 25**](#__RefHeading___Toc57189282)

[**Table S7 – Missing data for the UK Biobank population considered in one-sample Mendelian randomization investigating systolic blood pressure as an outcome. 26**](#__RefHeading___Toc57189283)

[**Table S8 – Variants and their weights for the urate genetic risk score used in one-sample Mendelian randomization. 27**](#__RefHeading___Toc57189284)

[**Table S9 – Characteristics of studies identified in the updated systematic review. 30**](#__RefHeading___Toc57189285)

[**Table S10 – Risk of bias for studies included in the systematic review and meta-analysis. 33**](#__RefHeading___Toc57189286)

[**Table S11 – Baseline, absolute change and relative change in serum urate concentration in the included clinical trials. 36**](#__RefHeading___Toc57189287)

[**Figure S1 – Scatter plot of the association for the instrument variants with serum urate and coronary heart disease (CHD) risk. 40**](#__RefHeading___Toc57189288)

[**Figure S2 – Scatter plot of the association for the instrument variants with serum urate and peripheral arterial disease (PAD) risk. 41**](#__RefHeading___Toc57189289)

[**Figure S3 – Scatter plot of the association for the instrument variants with serum urate and stroke risk. 42**](#__RefHeading___Toc57189290)

[**Figure S4 – Scatter plot of the association for the instrument variants with serum urate and systolic blood pressure (SBP). 43**](#__RefHeading___Toc57189291)

[**Figure S5 – Inverse-variance weighted (IVW) and multivariable Mendelian randomization (MVMR) estimates for the effect of 1-standard deviation (SD) increase in genetically determined serum urate levels on risk of coronary heart disease (CHD), peripheral artery disease (PAD) and stroke. The MVMR analysis adjusts for the association of the genetic instruments with systolic blood pressure, diastolic blood pressure and pulse pressure. 44**](#__RefHeading___Toc57189292)

[**Figure S6 – Inverse-variance weighted (IVW) and multivariable Mendelian randomization (MVMR) estimates for the effect of 1-standard deviation (SD) increase in genetically determined serum urate levels on risk of ischaemic stroke. The MVMR analysis adjusts for the association of the genetic instruments with systolic blood pressure. 45**](#__RefHeading___Toc57189293)

[**Figure S7 – Preferred Reporting Items for Systematic Reviews and Meta-Analyses (PRISMA) flow diagram. 46**](#__RefHeading___Toc57189294)

[**Figure S8 – Forest plot of randomized controlled trial estimates for change in mean systolic blood pressure in patients receiving urate-lowering therapy or placebo/no treatment. 47**](#__RefHeading___Toc57189295)

[**Figure S9 – Forest plot of randomized controlled trial with low risk of bias estimates for risk of major adverse cardiovascular events in all patients receiving urate-lowering therapy or placebo/no treatment in studies. 48**](#__RefHeading___Toc57189296)

[**Figure S10 – Forest plot of randomized controlled trial with low risk of bias estimates for risk of major adverse cardiovascular events in patients with existing cardiovascular disease receiving urate-lowering therapy or placebo/no treatment in studies. 49**](#__RefHeading___Toc57189297)

[**Figure S11 – Meta-regression analysis investigating the association of baseline systolic blood pressure (x-axis) with mean change in systolic blood pressure (y-axis) in randomized controlled trials of patients receiving urate-lowering therapy or placebo/no treatment. 50**](#__RefHeading___Toc57189298)

[**Figure S12 – Meta-regression analysis investigating the association of baseline serum urate concentration (x-axis) with mean change in systolic blood pressure (y-axis) in randomized controlled trials of patients receiving urate-lowering therapy or placebo/no treatment. 51**](#__RefHeading___Toc57189299)

## Supplementary Methods

### **Two-sample Mendelian randomization analysis**

#### **Statistical analysis**

Two-sample Mendelian randomization (MR) was used to estimate the causal effect of genetically determined serum urate levels on coronary heart disease (CHD), peripheral artery disease (PAD), stroke and systolic blood pressure (SBP). The inverse-variance weighted (IVW) approach was used for the main analysis. This calculates MR estimates for individual instrument single-nucleotide polymorphisms (SNPs) using the ratio method (and first-order weights) (1), with overall MR estimates derived by pooling these using random-effects IVW meta-analysis (2).

To explore the robustness of the MR estimates to potential pleiotropy of the genetic variants, where they affect the outcome of interest through pathways unrelated to the exposure under study, a series of statistical sensitivity analyses that are more robust to such pleiotropy were performed. Firstly, the contamination-mixture method was used (3). This method is based on the assumption that estimates derived from valid instruments follow a normal distribution centred on the true causal effect estimate, while those derived from invalid instruments follow a normal distribution centred on the null (3). This in turn allows for a likelihood function to be specified as a product of the two-component mixture distributions, with a profile likelihood approach used to maximize this function when allocating each variant to one of the mixture distributions (3). Secondly, MR-Egger was performed. This regresses the variant-outcome genetic association estimates on the variant-exposure genetic association estimates, weighted for the precision of the variant-outcome genetic association estimates (4). Provided that the association of the instrument variants with the exposure is not correlated to any direct effect (i.e. independent of the exposure) that they have on the outcome, then the slope of the regression offers an MR estimate with the intercept representing a test for directional pleiotropy (*P*<0.05) (4). Thirdly, the simple median estimator was applied (5). This orders the MR estimates derived from individual variants by their magnitude, selecting the median as the overall MR estimate, with standard error calculated by bootstrapping (5). Fourthly, the Pleiotropy Residual Sum and Outlier (PRESSO) method was also applied as an MR statistical sensitivity analysis (6). This regresses the variant-outcome association estimates by the variant-exposure association estimates and uses the residual errors to test for the presence of outlier variants. Any such outliers are removed and outlier-corrected MR estimates are consequently generated (6). Finally, regression-based multivariable MR was performed to investigate the effect of urate on the considered outcomes that is adjusted for any genetic confounding related to associations of the instrument variants to metabolic traits (7), namely BMI (8), estimated glomerular filtration rate (a measure of renal function) (9), type 2 diabetes mellitus (10), serum low-density lipoprotein cholesterol, high-density lipoprotein cholesterol and triglycerides (11). Publically-available summary genome-wide association study data related to the cited publications were used to identify instruments and obtain genetic association estimates for these metabolic traits. To select instruments for multivariable MR, all SNPs related to any of the considered exposure traits (including serum urate) at *P*<5x10^-8^ were pooled and clumped to pairwise linkage disequilibrium *r*^2^<0.001 (using the TwoSampleMR package of R (12)) based on the lowest *P*-value for association with any of the considered exposure traits. Multivariable MR was performed by regressing the variant-outcome association estimates by the variant-urate and various variant-metabolic trait estimates, weighted for the precision of the variant-outcome association, and with the intercept fixed to zero (13, 14).

All these MR sensitivity analyses were used to explore the robustness of the main IVW analysis, and as such no statistical significance thresholds were set for them, but rather the presence of consistent MR estimates was explored. The contamination-mixture, Egger, IVW and median MR analyses were performed using the MendelianRandomization R package (15), and PRESSO was performed using MRPRESSO R package (6).

In the presence of MR evidence to support an effect of serum urate on SBP, multivariable MR was performed to investigate for an effect of urate on the considered cardiovascular outcomes that was not mediated through SBP (16). The same approach for multivariable MR was used as described above, except that for this mediation analysis, only urate and SBP were included as exposures in the model. A threshold of *P*<0.05 was used to denote statistical significance of direct effect estimates in this analysis, as it was performed for the purpose of exploring for effects of urate on the respective cardiovascular outcomes that are not mediated through SBP.

### **One-sample Mendelian randomization analysis**

#### **Genetic association estimates**

One-sample MR analysis was performed in the UK Biobank, a prospective cohort study of approximately 500,000 participants recruited between 2006 and 2010 from 22 assessment centres across the UK (17). Self-reported, physical, biochemical and genetic information was collected and participants were linked retrospectively and prospectively to their hospital episode statistics as previously described (17). Analysis was restricted to self-reported White British participants. In order to increase statistical power while also maintaining consistency with the disease outcomes considered in the two-sample MR, CHD, PAD and stroke cases were pooled as CVD events. International Classification of Diseases version 10 codes were used to identify cases (Table S1). To obtain estimates for the association between the urate GRS and serum urate in UK Biobank participants, the score was regressed in a linear model against measured levels, with adjustment made for age, age^2^, sex, recruitment center and the first four principal components of genetic ancestry. To obtain genetic association estimates for the urate GRS with risk of incident CVD, a Cox proportional hazards regression was used, with adjustment similarly made for age, age^2^, sex, recruitment center and the first four principal components of genetic ancestry. Cases where death occurred prior to any incident CVD event were censored. Genetic association estimates of the urate GRS with SBP were obtained by performing a linear regression against SBP with the same adjustments as above. SBP was calculated as the mean of the two automated readings taken on the initial assessment, with 10mmHg added on for any antihypertensive medication use.

#### **Genetic variants used as instruments**

A weighted genetic risk score (GRS) for serum urate was used as the instrument in the one-sample MR analysis. This was constructed using SNPs with minor allele frequency >0.05 that were associated with urate at *P*<5x10^-8^ in the summary data from a GWAS performed in 288,666 European-ancestry individuals, clumped to a pairwise LD *r*^2^<0.001 using the TwoSampleMR package of R (7, 12). The UK Biobank population used to select instruments in the two-sample MR analysis was not included here to avoid overlap with the UK Biobank cohort used to obtain genetic association estimates with outcomes (18). Similarly, the population used to derive the weighted GRS for urate in the one-sample analysis was not also included when deriving instruments for the two-sample analysis because this would have resulted in overlap of the MVP population that was also used to obtain genetic association estimates for PAD (7, 18, 19). The GRS in the one-sample MR was weighted for the association of the constituent variants with serum urate, and for each individual participant was measured as the sum of the imputed number of alleles present multiplied by their variant’s weight.

#### **Statistical analysis**

To obtain MR estimates in the one-sample analysis, the ratio method was used, with first order weights for estimating standard errors (1). For consideration of sex-specific effects, the cohort was divided by self-reported sex. Exposure and outcome genetic association estimates for each sex were calculated separately. The Cochran’s Q test was used to investigate heterogeneity between males and females, with P<0.05 denoting statistical significance.

### **Systematic review and meta-analysis of randomized controlled trials**

#### **Literature search terms**

**PUBMED**

#7. #1 and (#2 or #3 or #4 or #5 or #6)

#6. Alopurinol or Allopurinol [Mesh] or Zyloprim or Wellcome Brand of Allopurinol or Allopurinol Wellcome Brand or Zyloric or Glaxo Wellcome Brand of Allopurinol or Allopurin or Bicther Brand of Allopurinol or Allopurinol Bicther Brand or Allorin or Douglas Brand of Allopurinol or Allopurinol Douglas Brand or Allpargin or Merz Brand of Allopurinol or Allopurinol Merz Brand or Allural or Pan Quimica or Quimica, Pan or Apulonga or Dorsch Brand of Allopurinol or Allopurinol Dorsch Brand or Apurin or Multipharma Brand of Allopurinol or Allopurinol Multipharma Brand or Atisuril or Byk Gulden Brand of Allopurinol or Bleminol or gepepharm Brand of Allopurinol or Allopurinol gepepharm Brand or Caplenal or Rhône-Poulenc Rorer Brand of Allopurinol or Rhône Poulenc Rorer Brand of Allopurinol or APS Brand of Allopurinol or Allopurinol APS Brand or Capurate or Fawns and McAllan Brand of Allopurinol or Cellidrin or Hennig Brand of Allopurinol or Allopurinol Hennig Brand or Embarin or Suspendol or Merckle Brand of Allopurinol or Allopurinol Merckle Brand or Foligan or Henning Berlin Brand of Allopurinol or Hamarin or Nicholas Brand of Allopurinol or Allopurinol Nicholas Brand or Jenapurinol or Jenapharm Brand of Allopurinol or Allopurinol Jenapharm Brand or Lopurin or Boots Brand of Allopurinol or Allopurinol Boots Brand or Lysuron or Boehringer Mannheim Brand of Allopurinol or Milurit or Thiemann Brand of Allopurinol or Allopurinol Thiemann Brand or Milurite or Novopurol or Novopharm Brand of Allopurinol or Allopurinol Novopharm Brand or Progout or Protea Brand of Allopurinol or Allopurinol Protea Brand or Alphapharm Brand of Allopurinol or Allopurinol Alphapharm Brand or Pureduct or Rosen Brand of Allopurinol or Allopurinol Rosen Brand or Purinol or Pinewood Brand of Allopurinol or Allopurinol Pinewood Brand or Horner Brand of Allopurinol or Allopurinol Horner Brand or Remid or TAD Brand of Allopurinol or Allopurinol TAD Brand or Rimapurinol or Rima Brand of Allopurinol or Allopurinol Rima Brand or Roucol or Rougier Brand of Allopurinol or Allopurinol Rougier Brand or Tipuric or Clonmel Brand of Allopurinol or Allopurinol Clonmel Brand or Uribenz or R.A.N. Brand of Allopurinol or Allopurinol R.A.N. Brand or Uridocid or Reig Jofre Brand of Allopurinol or Uripurinol or Azupharma Brand of Allopurinol or Allopurinol Azupharma Brand or Urosin or Roche Brand of Allopurinol or Allopurinol Roche Brand or Urtias or BASF Brand of Allopurinol or Allopurinol BASF Brand or Xanthomax or Ashbourne Brand of Allopurinol or Allopurinol Ashbourne Brand or Xanturic or Pharmafarm Brand of Allopurinol or Allopurinol Pharmafarm Brand or Zygout or Amrad Brand of Allopurinol or Allopurinol Amrad Brand or Allohexal or Hexal Brand of Allopurinol or Allopurinol Hexal Brand or Allohexan or Alloprin or ICN Brand of Allopurinol or Allopurinol ICN Brand

#5. febuxostat or "febuxostat" [Supplementary Concept] or “Uloric” or “2-(3-cyano-4-isobutoxyphenyl)-4-methyl-5-thiazolecarboxylic acid” or “TEI 6720” or “TEI-6720” or TEI6720

#4. "Oxypurinol"[Mesh] or Oxipurinol or Alloxanthine or tisopurine or "tisopurine"

[Supplementary Concept] or "thiopurinol" or "4-mercaptopyrazolo(3,4-d)pyrimidine" or topiroxostat or Uriadec or topiloric

#3. allosig or puricos or Ossipurinolo or Oxoallopurinol or Oxypurinolum or Alloxanthin or DHPP or Adenuric

#2. Xanthine Oxidase [Mesh] or Oxidase, Xanthine or Hypoxanthine Oxidase or Oxidase, Hypoxanthine or Purine-Xanthine Oxidase or Oxidase, Purine-Xanthine or Purine Xanthine Oxidase or Hypoxanthine Dehydrogenase or Dehydrogenase, Hypoxanthine or Hypoxanthine-Xanthine Oxidase or Hypoxanthine Xanthine Oxidase or Oxidase, Hypoxanthine-Xanthine or Xanthine Dehydrogenase [Mesh] or Dehydrogenase, Xanthine or Purine Hydroxylase I or Xanthine Oxidoreductase or Oxidoreductase, Xanthine

#1. (randomized controlled trial[pt] OR controlled clinical trial[pt] OR randomized controlled trials[mh] OR random allocation[mh] OR double-blind method[mh] OR single-blind method[mh] OR clinical trial[pt] OR clinical trials[mh] OR ("clinical trial"[tw]) OR ((singl*[tw] OR doubl*[tw] OR trebl*[tw] OR tripl*[tw]) AND (mask*[tw] OR blind*[tw])) OR ("latin square"[tw]) OR placebos[mh] OR placebo*[tw] OR random*[tw] OR research design[mh:noexp] OR follow-up studies[mh] OR prospective studies[mh] OR cross-over studies[mh] OR control*[tw] OR prospectiv*[tw] OR volunteer*[tw]) NOT (animal[mh] NOT human[mh])

**Web of Science**

TS=(clinical trial* OR research design OR comparative stud* OR evaluation stud* OR controlled trial* OR follow-up stud* OR prospective stud* OR random* OR placebo* OR single blind* OR double blind* OR triple blind* OR double dummy OR allocation)

TS=(alopurinol or allopurinol or Zyloprim or Zyloric or Allopurin or Allorin or Allpargin or Allural or "Pan Quimica" or "Quimica, Pan" or Apulonga or Apurin or Atisuril or Bleminol or Caplenal or Capurate or Cellidrin or Embarin or Suspendol or Foligan or Hamarin or Jenapurinol or Lopurin or Lysuron or Milurit or Milurite or Novopurol or Progout or Pureduct or Purinol or Remid or Rimapurinol or Roucol or Tipuric or Uribenz or Uridocid or Uripurinol or Urosin or Urtias or Xanthomax or Xanturic or Zygout or Allohexal or Allohexan or Alloprin or febuxostate or febuxostat or Uloric or "2-3-cyano-4-isobutoxyphenyl-4-methyl-5-thiazolecarboxylic acid" or "TEI 6720" or "TEI-6720" or TEI6720 or Oxypurinol or Oxipurinol or Alloxanthine or tisopurine or tisopurine or thiopurinol or "4-mercaptopyrazolo(3,4-d)pyrimidine" or topiroxostat or Uriadec or topiloric or allosig or puricos or Ossipurinolo or Oxoallopurinol or Oxypurinolum or Alloxanthin or DHPP or Adenuric or (Xanthine and oxidase) or (hypoxantine and oxidase) or (xanthine and dehydrogenase) or (hypoxanthine and dehydrogenase) or (xantina and oxidase) or (hipoxantina and oxidase) or (xantina and desidrogenase) or (hipoxantina and desidrogenase) or (xantina and oxidasa) or (hipoxantina and oxidasa) or (xanthine and oxidoreductase) or (hypoxanthine and oxidoreductase) or (xantina and oxiredutase) or (hipoxantina and oxiredutase) or (purine and hydroxylase))

**Embase**

1

(febuxostat exp or febuxostat).af.

2

(topiroxostat exp or topiroxostat).af.

3

(oxipurinol exp or oxipurinol or (oxypurinol exp or oxypurinol)).af.

4

(allosig or allosig exp or allosig or puricos or puricos or puricos or ossipurinolo or oxoallopurinol or oxypurinolum or alloxanthin or alloxanthin exp or alloxanthin or dhpp or adenuric or adenuric exp or adenuric).af.

5

(xanthine oxidase exp or xanthine oxidase).af.

6

(allopurinol exp or allopurinol).af.

7

(((((crossover procedure exp or crossover procedure or prospective study exp or prospective study or follow up exp or follow up or placebo exp or placebo or clinical trial exp or clinical trial or single blind procedure exp or single blind procedure or double blind procedure exp or double blind procedure or triple blind procedure exp or triple blind procedure or randomization exp or randomization or controlled clinical trial exp or controlled clinical trial or randomized controlled trial exp or randomized controlled trial) not animals exp) or animals) not humans exp) or humans).af.

8

1 or 2 or 3 or 4 or 5 or 6

9

7 and 8

10

9 and 2016:2019.(sa_year).

11

10 and 2016:2019.(sa_year).

**Cochrane**

(randomized controlled trial OR controlled clinical trial OR randomized controlled trials OR random allocation OR double-blind method OR single-blind method OR clinical trial OR clinical trials OR clinical trial OR (singl* OR doubl* OR trebl* OR tripl*) AND (mask OR blind*) OR ("latin square") OR placebos OR placebo* OR random* OR research design OR follow-up studies OR prospective studies OR cross-over studies OR control* OR prospectiv* OR volunteer*) NOT (animal NOT human)

Xanthine Oxidase or Oxidase, Xanthine or Hypoxanthine Oxidase or Oxidase, Hypoxanthine or Purine-Xanthine Oxidase or Oxidase, Purine-Xanthine or Purine Xanthine Oxidase or Hypoxanthine Dehydrogenase or Dehydrogenase, Hypoxanthine or Hypoxanthine-Xanthine Oxidase or Hypoxanthine Xanthine Oxidase or Oxidase, Hypoxanthine-Xanthine or Xanthine Dehydrogenase or Dehydrogenase, Xanthine or Purine Hydroxylase I or Xanthine Oxidoreductase or Oxidoreductase, Xanthine

allosig or puricos or Ossipurinolo or Oxoallopurinol or Oxypurinolum or Alloxanthin or DHPP or Adenuric or "Oxypurinol" or Oxipurinol or Alloxanthine or tisopurine or "tisopurine" or "thiopurinol" or "4-mercaptopyrazolo(3,4-d)pyrimidine" or topiroxostat or Uriadec or topiloric

(febuxostat or febuxostat or Uloric or 2-3-cyano-4-isobutoxyphenyl-4-methyl-5-thiazolecarboxylic acid or TEI 6720 or TEI-6720 or TEI6720)

Alopurinol or Allopurinol or Zyloprim or Wellcome Brand of Allopurinol or Allopurinol Wellcome Brand or Zyloric or Glaxo Wellcome Brand of Allopurinol or Allopurin or Bicther Brand of Allopurinol or Allopurinol Bicther Brand or Allorin or Douglas Brand of Allopurinol or Allopurinol Douglas Brand or Allpargin or Merz Brand of Allopurinol or Allopurinol Merz Brand or Allural or Pan Quimica or Quimica, Pan or Apulonga or Dorsch Brand of Allopurinol or Allopurinol Dorsch Brand or Apurin or Multipharma Brand of Allopurinol or Allopurinol Multipharma Brand or Atisuril or Byk Gulden Brand of Allopurinol or Bleminol or gepepharm Brand of Allopurinol or Allopurinol gepepharm Brand or Caplenal or Rhône-Poulenc Rorer Brand of Allopurinol or Rhône Poulenc Rorer Brand of Allopurinol or APS Brand of Allopurinol or Allopurinol APS Brand or Capurate or Fawns and McAllan Brand of Allopurinol or Cellidrin or Hennig Brand of Allopurinol or Allopurinol Hennig Brand or Embarin or Suspendol or Merckle Brand of Allopurinol or Allopurinol Merckle Brand or Foligan or Henning Berlin Brand of Allopurinol or Hamarin or Nicholas Brand of Allopurinol or Allopurinol Nicholas Brand or Jenapurinol or Jenapharm Brand of Allopurinol or Allopurinol Jenapharm Brand or Lopurin or Boots Brand of Allopurinol or Allopurinol Boots Brand or Lysuron or Boehringer Mannheim Brand of Allopurinol or Milurit or Thiemann Brand of Allopurinol or Allopurinol Thiemann Brand or Milurite or Novopurol or Novopharm Brand of Allopurinol or Allopurinol Novopharm Brand or Progout or Protea Brand of Allopurinol or Allopurinol Protea Brand or Alphapharm Brand of Allopurinol or Allopurinol Alphapharm Brand or Pureduct or Rosen Brand of Allopurinol or Allopurinol Rosen Brand or Purinol or Pinewood Brand of Allopurinol or Allopurinol Pinewood Brand or Horner Brand of Allopurinol or Allopurinol Horner Brand or Remid or TAD Brand of Allopurinol or Allopurinol TAD Brand or Rimapurinol or Rima Brand of Allopurinol or Allopurinol Rima Brand or Roucol or Rougier Brand of Allopurinol or Allopurinol Rougier Brand or Tipuric or Clonmel Brand of Allopurinol or Allopurinol Clonmel Brand or Uribenz or R.A.N. Brand of Allopurinol or Allopurinol R.A.N. Brand or Uridocid or Reig Jofre Brand of Allopurinol or Uripurinol or Azupharma Brand of Allopurinol or Allopurinol Azupharma Brand or Urosin or Roche Brand of Allopurinol or Allopurinol Roche Brand or Urtias or BASF Brand of Allopurinol or Allopurinol BASF Brand or Xanthomax or Ashbourne Brand of Allopurinol or Allopurinol Ashbourne Brand or Xanturic or Pharmafarm Brand of Allopurinol or Allopurinol Pharmafarm Brand or Zygout or Amrad Brand of Allopurinol or Allopurinol Amrad Brand or Allohexal or Hexal Brand of Allopurinol or Allopurinol Hexal Brand or Allohexan or Alloprin or ICN Brand of Allopurinol or Allopurinol ICN Brand

## Supplementary Results

### **One-sample Mendelian randomization**

Baseline characteristics for the UK Biobank population considered in the one-sample MR analysis are detailed in Table S6. A total of 392,282 participants were included in the analysis for SBP after accounting for missing data (Table S7). Included participants had similar characteristics to the UK Biobank population more generally (Table S6). The first participant was recruited on 13 March 2006 and outcome data were available up to 31 March 2017. A total of 34,538 incident CVD events (30,314 coronary heart disease, 467 peripheral arterial disease and 3,757 stroke) were identified in the analysis, with a mean follow up time of 2,865 days. The 107 SNPs included in the urate GRS for the one-sample MR and their respective weights are detailed in Table S8. Considering the whole population, the OR of CVD per 1-SD increase in genetically predicted urate was 1.13 (95% CI 1.07-1.19, *P*=1x10^-6^) and the SD change in SBP per 1-SD increase in genetically predicted urate was 0.06 (95% CI 0.05-0.07, *P*=3x10^-18^). When stratifying by sex, the corresponding estimates were 1.15 (95% CI 1.08-1.23, *P*=2x10^-5^) for CVD and 0.05 (95% CI 0.03-0.07, *P*=2x10^-5^) for SBP in males, and 1.10 (95% CI 1.01-1.19, *P*=0.02) for CVD and 0.08 (95% CI 0.06-0.09, *P*=2x10^-15^) for SBP in females. There was no evidence of heterogeneity between males and females for CVD risk (*P*=0.37), but weak evidence of heterogeneity for SBP (*P*=0.04).

## References

1. Palmer TM, Sterne JA, Harbord RM, Lawlor DA, Sheehan NA, Meng S, et al. Instrumental variable estimation of causal risk ratios and causal odds ratios in Mendelian randomization analyses. Am J Epidemiol. 2011;173(12):1392-403.

2. Burgess S, Butterworth A, Thompson SG. Mendelian randomization analysis with multiple genetic variants using summarized data. Genet Epidemiol. 2013;37(7):658-65.

3. Burgess S, Foley CN, Allara E, Staley JR, Howson JM. A robust and efficient method for Mendelian randomization with hundreds of genetic variants: unravelling mechanisms linking HDL-cholesterol and coronary heart disease. bioRxiv. 2019:566851.

4. Bowden J, Davey Smith G, Burgess S. Mendelian randomization with invalid instruments: effect estimation and bias detection through Egger regression. Int J Epidemiol. 2015;44(2):512-25.

5. Bowden J, Davey Smith G, Haycock PC, Burgess S. Consistent Estimation in Mendelian Randomization with Some Invalid Instruments Using a Weighted Median Estimator. Genet Epidemiol. 2016;40(4):304-14.

6. Verbanck M, Chen CY, Neale B, Do R. Detection of widespread horizontal pleiotropy in causal relationships inferred from Mendelian randomization between complex traits and diseases. Nat Genet. 2018;50(5):693-8.

7. Tin A, Marten J, Halperin Kuhns VL, Li Y, Wuttke M, Kirsten H, et al. Target genes, variants, tissues and transcriptional pathways influencing human serum urate levels. Nat Genet. 2019.

8. Yengo L, Sidorenko J, Kemper KE, Zheng Z, Wood AR, Weedon MN, et al. Meta-analysis of genome-wide association studies for height and body mass index in approximately 700000 individuals of European ancestry. Hum Mol Genet. 2018;27(20):3641-9.

9. Wuttke M, Li Y, Li M, Sieber KB, Feitosa MF, Gorski M, et al. A catalog of genetic loci associated with kidney function from analyses of a million individuals. Nat Genet. 2019;51(6):957-72.

10. Mahajan A, Taliun D, Thurner M, Robertson NR, Torres JM, Rayner NW, et al. Fine-mapping type 2 diabetes loci to single-variant resolution using high-density imputation and islet-specific epigenome maps. Nat Genet. 2018;50(11):1505-13.

11. Willer CJ, Schmidt EM, Sengupta S, Peloso GM, Gustafsson S, Kanoni S, et al. Discovery and refinement of loci associated with lipid levels. Nat Genet. 2013;45(11):1274-83.

12. Hemani G, Zheng J, Elsworth B, Wade KH, Haberland V, Baird D, et al. The MR-Base platform supports systematic causal inference across the human phenome. eLife. 2018;7.

13. Burgess S, Dudbridge F, Thompson SG. Re: "Multivariable Mendelian randomization: the use of pleiotropic genetic variants to estimate causal effects". Am J Epidemiol. 2015;181(4):290-1.

14. Burgess S, Freitag DF, Khan H, Gorman DN, Thompson SG. Using multivariable Mendelian randomization to disentangle the causal effects of lipid fractions. PLoS One. 2014;9(10):e108891.

15. Yavorska OO, Burgess S. MendelianRandomization: an R package for performing Mendelian randomization analyses using summarized data. Int J Epidemiol. 2017;46(6):1734-9.

16. Burgess S, Thompson DJ, Rees JMB, Day FR, Perry JR, Ong KK. Dissecting Causal Pathways Using Mendelian Randomization with Summarized Genetic Data: Application to Age at Menarche and Risk of Breast Cancer. Genetics. 2017;207(2):481-7.

17. Sudlow C, Gallacher J, Allen N, Beral V, Burton P, Danesh J, et al. UK Biobank: An Open Access Resource for Identifying the Causes of a Wide Range of Complex Diseases of Middle and Old Age. PLoS Med. 2015;12(3):e1001779.

18. Burgess S, Davies NM, Thompson SG. Bias due to participant overlap in two-sample Mendelian randomization. Genet Epidemiol. 2016;40(7):597-608.

19. Klarin D, Lynch J, Aragam K, Chaffin M, Assimes TL, Huang J, et al. Genome-wide association study of peripheral artery disease in the Million Veteran Program. Nat Med. 2019;25(8):1274-9.

20. Alshahawey M, Shahin SM, Elsaid TW, Sabri NA. Effect of Febuxostat on the Endothelial Dysfunction in Hemodialysis Patients: A Randomized, Placebo-Controlled, Double-Blinded Study. Am J Nephrol. 2017;45(5):452-9.

21. Dalbeth N, Saag KG, Palmer WE, Choi HK, Hunt B, MacDonald PA, et al. Effects of Febuxostat in Early Gout: A Randomized, Double-Blind, Placebo-Controlled Study. Arthritis Rheumatol. 2017;69(12):2386-95.

22. Gingles CR, Symon R, Gandy SJ, Struthers AD, Houston G, MacDonald TM, et al. Allopurinol treatment adversely impacts left ventricular mass regression in patients with well-controlled hypertension. J Hypertens. 2019;37(12):2481-9.

23. Gunawardhana L, McLean L, Punzi HA, Hunt B, Palmer RN, Whelton A, et al. Effect of Febuxostat on Ambulatory Blood Pressure in Subjects With Hyperuricemia and Hypertension: A Phase 2 Randomized Placebo-Controlled Study. J Am Heart Assoc. 2017;6(11).

24. Huang Y, Zhang C, Xu Z, Shen J, Zhang X, Du H, et al. Clinical Study on efficacy of allopurinol in patients with acute coronary syndrome and its functional mechanism. Hellenic J Cardiol. 2017;58(5):360-5.

25. Kimura K, Hosoya T, Uchida S, Inaba M, Makino H, Maruyama S, et al. Febuxostat Therapy for Patients With Stage 3 CKD and Asymptomatic Hyperuricemia: A Randomized Trial. Am J Kidney Dis. 2018;72(6):798-810.

26. McMullan CJ, Borgi L, Fisher N, Curhan G, Forman J. Effect of Uric Acid Lowering on Renin-Angiotensin-System Activation and Ambulatory BP: A Randomized Controlled Trial. Clin J Am Soc Nephrol. 2017;12(5):807-16.

27. Morgan BJ, Teodorescu M, Pegelow DF, Jackson ER, Schneider DL, Plante DT, et al. Effects of losartan and allopurinol on cardiorespiratory regulation in obstructive sleep apnoea. Exp Physiol. 2018;103(7):941-55.

28. Mukri MNA, Kong WY, Mustafar R, Shaharir SS, Shah SA, Abdul Gafor AH, et al. Role of febuxostat in retarding progression of diabetic kidney disease with asymptomatic hyperuricemia: A 6-months open-label, randomized controlled trial. EXCLI J. 2018;17:563-75.

29. Omrani H, Sadeghi S, Raeisi D, Hashemian A. The effect of allopurinol treatment regimen on serum uric acid and arterial blood pressure in haemodialysis patients. J Kermanshah Univ Med Sci. 2016;20(2):56-61.

30. Saag KG, Becker MA, Whelton A, Hunt B, Castillo M, Kisfalvi K, et al. Efficacy and Safety of Febuxostat Extended and Immediate Release in Patients With Gout and Renal Impairment: A Phase III Placebo-Controlled Study. Arthritis Rheumatol. 2019;71(1):143-53.

31. Tausche AK, Alten R, Dalbeth N, Kopicko J, Fung M, Adler S, et al. Lesinurad monotherapy in gout patients intolerant to a xanthine oxidase inhibitor: a 6 month phase 3 clinical trial and extension study. Rheumatology (Oxford). 2017;56(12):2170-8.

32. Wada T, Hosoya T, Honda D, Sakamoto R, Narita K, Sasaki T, et al. Uric acid-lowering and renoprotective effects of topiroxostat, a selective xanthine oxidoreductase inhibitor, in patients with diabetic nephropathy and hyperuricemia: a randomized, double-blind, placebo-controlled, parallel-group study (UPWARD study). Clin Exp Nephrol. 2018;22(4):860-70.

## Table S1 – International Classification of Diseases version 10 (ICD-10) codes used to identify incident cardiovascular disease events

| **Disease category** | **ICD-10 code** |
| --- | --- |
| Coronary heart disease | I20-I25 |
| Stroke | I61, I63, I64 |
| Peripheral arterial disease | I70.0, I70.2-I70.7, I70.9, I73.9 |
| Cardiovascular disease | All of the above |

## Table S2 – Two-sample Mendelian randomization instruments for urate

| **Chromosome** | **Position (hg19)** | **SNP** | **Allele1** | **Allele2** | **Allele1 frequency** | **Beta** | **Standard error** | **P** | **R^2^** | **F** |
| --- | --- | --- | --- | --- | --- | --- | --- | --- | --- | --- |
| 1 | 1087683 | rs9442380 | T | C | 0.07 | -2.65E-02 | 3.76E-03 | 8.83E-13 | 8.96E-05 | 41 |
| 1 | 2156362 | rs2460000 | A | G | 0.34 | -1.28E-02 | 1.98E-03 | 5.58E-11 | 7.32E-05 | 33 |
| 1 | 15876758 | rs10803392 | T | C | 0.31 | -2.07E-02 | 1.98E-03 | 9.03E-26 | 1.84E-04 | 84 |
| 1 | 20158241 | rs4509550 | T | C | 0.60 | 1.10E-02 | 1.91E-03 | 3.58E-09 | 5.83E-05 | 26 |
| 1 | 27019744 | rs12738345 | T | C | 0.92 | -3.19E-02 | 3.39E-03 | 2.51E-21 | 1.56E-04 | 71 |
| 1 | 46086329 | rs11590549 | T | C | 0.24 | 1.17E-02 | 2.18E-03 | 3.97E-08 | 5.02E-05 | 23 |
| 1 | 48685435 | rs926979 | T | C | 0.33 | -1.38E-02 | 1.98E-03 | 1.87E-12 | 8.33E-05 | 38 |
| 1 | 51086337 | rs10888700 | T | C | 0.57 | 1.73E-02 | 1.84E-03 | 3.49E-21 | 1.47E-04 | 67 |
| 1 | 82944571 | rs10874312 | A | G | 0.66 | 1.13E-02 | 1.94E-03 | 2.85E-09 | 5.77E-05 | 26 |
| 1 | 93837133 | rs12752223 | T | C | 0.40 | -1.75E-02 | 1.88E-03 | 4.29E-21 | 1.47E-04 | 67 |
| 1 | 110147321 | rs17024258 | T | C | 0.03 | 3.31E-02 | 5.78E-03 | 5.09E-09 | 5.52E-05 | 25 |
| 1 | 120264573 | rs3790707 | T | C | 0.02 | 3.58E-02 | 6.47E-03 | 1.57E-08 | 5.31E-05 | 24 |
| 1 | 145723739 | rs1471633 | A | C | 0.46 | 4.13E-02 | 1.83E-03 | 6.82E-114 | 8.50E-04 | 386 |
| 1 | 155155731 | rs4971100 | A | G | 0.43 | -3.60E-02 | 1.87E-03 | 1.15E-82 | 6.33E-04 | 288 |
| 1 | 163741788 | rs2345962 | A | G | 0.50 | -1.47E-02 | 1.84E-03 | 5.53E-16 | 1.09E-04 | 49 |
| 1 | 186762079 | rs16825911 | C | G | 0.02 | -3.89E-02 | 5.85E-03 | 1.46E-11 | 7.28E-05 | 33 |
| 1 | 200251050 | rs4915419 | A | C | 0.59 | -1.16E-02 | 1.90E-03 | 4.05E-10 | 6.57E-05 | 30 |
| 1 | 201793440 | rs2644128 | C | G | 0.45 | -1.30E-02 | 1.85E-03 | 9.97E-13 | 8.36E-05 | 38 |
| 1 | 205031769 | rs2242000 | A | G | 0.18 | 1.58E-02 | 2.41E-03 | 3.07E-11 | 7.30E-05 | 33 |
| 1 | 212069929 | rs2788144 | A | G | 0.96 | -3.71E-02 | 4.95E-03 | 3.19E-14 | 9.76E-05 | 44 |
| 1 | 227170298 | rs2297412 | A | G | 0.52 | -1.09E-02 | 1.94E-03 | 9.27E-09 | 5.92E-05 | 27 |
| 1 | 234863602 | rs478425 | T | G | 0.37 | -1.34E-02 | 1.91E-03 | 1.40E-12 | 8.34E-05 | 38 |
| 2 | 651407 | rs12714414 | T | C | 0.85 | 1.70E-02 | 2.62E-03 | 4.62E-11 | 7.43E-05 | 34 |
| 2 | 15782471 | rs807624 | T | G | 0.36 | -1.77E-02 | 1.91E-03 | 1.01E-20 | 1.44E-04 | 65 |
| 2 | 18677222 | rs12105304 | A | G | 0.68 | -2.02E-02 | 1.95E-03 | 2.36E-25 | 1.77E-04 | 80 |
| 2 | 27730940 | rs1260326 | T | C | 0.39 | 5.13E-02 | 1.86E-03 | 1.34E-167 | 1.26E-03 | 571 |
| 2 | 32679732 | rs3769598 | A | G | 0.86 | 1.81E-02 | 2.65E-03 | 4.31E-12 | 7.99E-05 | 36 |
| 2 | 50692168 | rs1520455 | T | C | 0.61 | -1.06E-02 | 1.98E-03 | 4.64E-08 | 5.34E-05 | 24 |
| 2 | 61494450 | rs4671402 | A | C | 0.52 | 1.21E-02 | 1.85E-03 | 2.71E-11 | 7.33E-05 | 33 |
| 2 | 69821008 | rs11693363 | A | C | 0.87 | 2.88E-02 | 2.75E-03 | 4.13E-26 | 1.89E-04 | 86 |
| 2 | 85703308 | rs6714421 | A | G | 0.61 | 1.12E-02 | 1.92E-03 | 2.91E-09 | 5.95E-05 | 27 |
| 2 | 101627925 | rs1062062 | T | C | 0.14 | 1.65E-02 | 2.64E-03 | 2.31E-10 | 6.60E-05 | 30 |
| 2 | 111932997 | rs6760053 | C | G | 0.53 | 1.10E-02 | 1.91E-03 | 4.68E-09 | 5.97E-05 | 27 |
| 2 | 121306440 | rs17050272 | A | G | 0.41 | 2.56E-02 | 1.89E-03 | 4.90E-42 | 3.18E-04 | 144 |
| 2 | 122003073 | rs882759 | A | G | 0.64 | 1.42E-02 | 1.95E-03 | 1.54E-13 | 9.34E-05 | 42 |
| 2 | 134511570 | rs1900351 | A | G | 0.37 | -1.05E-02 | 1.92E-03 | 2.14E-08 | 5.13E-05 | 23 |
| 2 | 136603366 | rs3769005 | C | G | 0.20 | -1.34E-02 | 2.26E-03 | 1.72E-09 | 5.73E-05 | 26 |
| 2 | 148627528 | rs929939 | A | C | 0.30 | 2.05E-02 | 1.98E-03 | 2.13E-25 | 1.77E-04 | 81 |
| 2 | 170033995 | rs7565788 | T | C | 0.25 | 2.60E-02 | 2.12E-03 | 9.38E-35 | 2.50E-04 | 114 |
| 2 | 170187105 | rs3845731 | A | G | 0.21 | 1.68E-02 | 2.26E-03 | 5.24E-14 | 9.34E-05 | 42 |
| 2 | 176951487 | rs12474905 | T | C | 0.20 | 1.39E-02 | 2.35E-03 | 1.89E-09 | 6.24E-05 | 28 |
| 2 | 183086648 | rs6741055 | C | G | 0.51 | -1.53E-02 | 1.84E-03 | 5.21E-17 | 1.16E-04 | 53 |
| 2 | 203255485 | rs13426118 | A | C | 0.88 | -2.53E-02 | 2.81E-03 | 9.20E-20 | 1.36E-04 | 62 |
| 2 | 211543055 | rs715 | T | C | 0.69 | 2.40E-02 | 2.03E-03 | 1.32E-32 | 2.48E-04 | 113 |
| 2 | 213373521 | rs1384291 | T | C | 0.16 | -2.03E-02 | 2.53E-03 | 5.20E-16 | 1.09E-04 | 49 |
| 2 | 227105921 | rs2943650 | T | C | 0.65 | 1.23E-02 | 1.94E-03 | 1.02E-10 | 6.97E-05 | 32 |
| 2 | 242421866 | rs871375 | A | G | 0.66 | -1.67E-02 | 1.94E-03 | 3.22E-18 | 1.26E-04 | 57 |
| 3 | 12385828 | rs11128603 | A | G | 0.88 | 2.18E-02 | 2.89E-03 | 2.67E-14 | 1.01E-04 | 46 |
| 3 | 12622623 | rs9849171 | C | G | 0.36 | -1.21E-02 | 1.93E-03 | 1.79E-10 | 6.74E-05 | 31 |
| 3 | 15846066 | rs13075443 | A | G | 0.66 | -1.11E-02 | 2.00E-03 | 1.41E-08 | 5.58E-05 | 25 |
| 3 | 49878652 | rs1317140 | A | G | 0.32 | 1.55E-02 | 1.97E-03 | 2.19E-15 | 1.04E-04 | 47 |
| 3 | 53062661 | rs9847710 | T | C | 0.58 | -3.33E-02 | 1.85E-03 | 3.39E-72 | 5.40E-04 | 246 |
| 3 | 69082657 | rs937859 | T | C | 0.65 | 1.50E-02 | 1.94E-03 | 5.65E-15 | 1.02E-04 | 46 |
| 3 | 94039386 | rs4857338 | T | G | 0.56 | -1.16E-02 | 1.86E-03 | 2.50E-10 | 6.59E-05 | 30 |
| 3 | 114444572 | rs11711620 | A | C | 0.83 | 1.41E-02 | 2.50E-03 | 8.56E-09 | 5.57E-05 | 25 |
| 3 | 126012852 | rs6804946 | T | C | 0.80 | 2.00E-02 | 2.30E-03 | 1.57E-18 | 1.28E-04 | 58 |
| 3 | 132222601 | rs7651369 | A | G | 0.65 | 1.12E-02 | 1.94E-03 | 3.53E-09 | 5.75E-05 | 26 |
| 3 | 141682825 | rs3792409 | C | G | 0.74 | 2.09E-02 | 2.09E-03 | 1.03E-23 | 1.69E-04 | 77 |
| 3 | 142748718 | rs4030582 | T | C | 0.63 | -1.06E-02 | 1.92E-03 | 1.86E-08 | 5.24E-05 | 24 |
| 3 | 153885006 | rs357488 | A | G | 0.15 | -1.58E-02 | 2.64E-03 | 1.05E-09 | 6.28E-05 | 29 |
| 3 | 156919138 | rs16827308 | T | G | 0.30 | 1.09E-02 | 2.03E-03 | 3.90E-08 | 4.94E-05 | 22 |
| 3 | 168788692 | rs1479404 | T | C | 0.55 | 9.93E-03 | 1.86E-03 | 4.73E-08 | 4.89E-05 | 22 |
| 3 | 169091584 | rs1290791 | A | G | 0.74 | 1.75E-02 | 2.09E-03 | 2.85E-17 | 1.18E-04 | 53 |
| 3 | 170670279 | rs16855567 | T | C | 0.08 | 1.97E-02 | 3.45E-03 | 6.17E-09 | 5.46E-05 | 25 |
| 3 | 185296602 | rs11918734 | T | C | 0.74 | -1.25E-02 | 2.10E-03 | 1.21E-09 | 6.10E-05 | 28 |
| 4 | 9722275 | rs13121465 | T | G | 0.34 | -7.66E-02 | 1.94E-03 | <1E-300 | 2.63E-03 | 1198 |
| 4 | 10104788 | rs717614 | C | G | 0.52 | 1.44E-01 | 1.81E-03 | <1E-300 | 1.04E-02 | 4758 |
| 4 | 10153233 | rs737601 | T | C | 0.92 | -4.94E-02 | 3.36E-03 | 3.34E-49 | 3.63E-04 | 165 |
| 4 | 10704137 | rs13120565 | A | T | 0.38 | 2.66E-02 | 1.92E-03 | 5.91E-44 | 3.33E-04 | 151 |
| 4 | 22820504 | rs358231 | A | T | 0.15 | -1.78E-02 | 2.58E-03 | 3.10E-12 | 7.85E-05 | 36 |
| 4 | 39414993 | rs11940694 | A | G | 0.39 | -1.03E-02 | 1.92E-03 | 4.25E-08 | 5.06E-05 | 23 |
| 4 | 87790805 | rs13103212 | T | C | 0.08 | -1.81E-02 | 3.37E-03 | 3.62E-08 | 5.07E-05 | 23 |
| 4 | 88932272 | rs2725234 | T | C | 0.10 | 1.16E-01 | 3.04E-03 | <1E-300 | 2.48E-03 | 1128 |
| 4 | 89039500 | rs2231146 | T | C | 0.99 | 6.23E-02 | 9.72E-03 | 7.31E-11 | 7.70E-05 | 35 |
| 4 | 89119659 | rs10856870 | T | C | 0.45 | -3.86E-02 | 1.85E-03 | 1.90E-97 | 7.38E-04 | 336 |
| 4 | 89453522 | rs2869665 | A | G | 0.78 | -1.54E-02 | 2.29E-03 | 8.19E-12 | 8.15E-05 | 37 |
| 4 | 103188709 | rs13107325 | T | C | 0.07 | -2.97E-02 | 3.54E-03 | 2.58E-17 | 1.22E-04 | 55 |
| 4 | 144140665 | rs11724872 | T | C | 0.60 | -1.52E-02 | 1.87E-03 | 2.38E-16 | 1.11E-04 | 50 |
| 4 | 146722152 | rs6854834 | A | G | 0.14 | -1.68E-02 | 2.68E-03 | 1.77E-10 | 6.75E-05 | 31 |
| 4 | 146831323 | rs12510175 | C | G | 0.66 | 1.20E-02 | 1.96E-03 | 4.98E-10 | 6.46E-05 | 29 |
| 5 | 34657025 | rs461660 | A | C | 0.54 | 1.80E-02 | 1.86E-03 | 1.70E-22 | 1.61E-04 | 73 |
| 5 | 40615122 | rs924967 | C | G | 0.12 | -1.59E-02 | 2.89E-03 | 1.92E-08 | 5.21E-05 | 24 |
| 5 | 52783408 | rs1469101 | T | C | 0.60 | -1.04E-02 | 1.90E-03 | 2.59E-08 | 5.14E-05 | 23 |
| 5 | 53319517 | rs255751 | T | C | 0.70 | 1.82E-02 | 1.99E-03 | 2.77E-20 | 1.40E-04 | 63 |
| 5 | 55450211 | rs10077826 | T | C | 0.28 | -1.31E-02 | 2.13E-03 | 4.23E-10 | 6.83E-05 | 31 |
| 5 | 55806751 | rs459193 | A | G | 0.25 | -2.10E-02 | 2.19E-03 | 4.16E-22 | 1.67E-04 | 76 |
| 5 | 67713205 | rs10075612 | T | G | 0.28 | 1.37E-02 | 2.06E-03 | 1.90E-11 | 7.47E-05 | 34 |
| 5 | 68648770 | rs9291949 | A | G | 0.47 | -1.02E-02 | 1.87E-03 | 2.39E-08 | 5.21E-05 | 24 |
| 5 | 72431482 | rs17632159 | C | G | 0.30 | -3.23E-02 | 2.00E-03 | 6.48E-59 | 4.42E-04 | 201 |
| 5 | 90226126 | rs3105792 | T | G | 0.32 | -1.29E-02 | 1.98E-03 | 4.74E-11 | 7.14E-05 | 32 |
| 5 | 131590534 | rs7727544 | T | C | 0.56 | 1.36E-02 | 1.85E-03 | 1.17E-13 | 9.08E-05 | 41 |
| 5 | 173298226 | rs6864880 | T | C | 0.31 | -1.29E-02 | 2.02E-03 | 7.24E-11 | 7.19E-05 | 33 |
| 5 | 176780545 | rs4131289 | A | G | 0.31 | 1.75E-02 | 1.99E-03 | 6.72E-19 | 1.31E-04 | 59 |
| 6 | 2064648 | rs676015 | T | C | 0.37 | 1.40E-02 | 1.93E-03 | 1.73E-13 | 9.14E-05 | 42 |
| 6 | 6727363 | rs1294448 | C | G | 0.50 | -1.30E-02 | 1.94E-03 | 1.01E-11 | 8.45E-05 | 38 |
| 6 | 7102084 | rs675209 | T | C | 0.27 | 4.01E-02 | 2.06E-03 | 1.05E-84 | 6.32E-04 | 287 |
| 6 | 7122936 | rs1297769 | A | G | 0.05 | 2.28E-02 | 4.24E-03 | 3.60E-08 | 5.19E-05 | 24 |
| 6 | 25813150 | rs1165196 | A | G | 0.57 | 6.72E-02 | 1.83E-03 | 5.41E-295 | 2.21E-03 | 1007 |
| 6 | 27377668 | rs12525255 | T | C | 0.39 | 1.15E-02 | 1.90E-03 | 8.39E-10 | 6.27E-05 | 28 |
| 6 | 29695854 | rs2735059 | A | G | 0.43 | -1.36E-02 | 1.89E-03 | 2.72E-13 | 9.09E-05 | 41 |
| 6 | 31544189 | rs3093662 | A | G | 0.92 | 2.23E-02 | 3.55E-03 | 1.66E-10 | 6.96E-05 | 32 |
| 6 | 39136651 | rs12214118 | A | G | 0.79 | 1.55E-02 | 2.27E-03 | 4.53E-12 | 8.00E-05 | 36 |
| 6 | 43805362 | rs744103 | A | T | 0.69 | 3.18E-02 | 2.00E-03 | 2.14E-57 | 4.37E-04 | 198 |
| 6 | 44030011 | rs833805 | A | G | 0.12 | -2.64E-02 | 3.17E-03 | 3.94E-17 | 1.42E-04 | 64 |
| 6 | 50963777 | rs4711966 | A | C | 0.34 | 1.09E-02 | 1.96E-03 | 1.33E-08 | 5.34E-05 | 24 |
| 6 | 55593250 | rs9475390 | T | C | 0.07 | 2.15E-02 | 3.69E-03 | 3.02E-09 | 5.88E-05 | 27 |
| 6 | 90127390 | rs854917 | T | C | 0.74 | -1.28E-02 | 2.14E-03 | 1.13E-09 | 6.32E-05 | 29 |
| 6 | 111548813 | rs354532 | T | C | 0.54 | 1.03E-02 | 1.87E-03 | 1.72E-08 | 5.27E-05 | 24 |
| 6 | 116232098 | rs195485 | T | C | 0.38 | -1.38E-02 | 1.91E-03 | 2.12E-13 | 9.06E-05 | 41 |
| 6 | 126228512 | rs10782230 | A | G | 0.48 | 1.51E-02 | 1.85E-03 | 1.51E-16 | 1.14E-04 | 52 |
| 6 | 133934829 | rs2677829 | A | G | 0.61 | 1.18E-02 | 1.90E-03 | 2.17E-10 | 6.64E-05 | 30 |
| 6 | 160771363 | rs505870 | A | C | 0.48 | -1.20E-02 | 1.85E-03 | 4.50E-11 | 7.21E-05 | 33 |
| 7 | 1285195 | rs10277115 | A | T | 0.24 | -3.07E-02 | 2.31E-03 | 1.58E-40 | 3.43E-04 | 156 |
| 7 | 4708134 | rs17828856 | A | G | 0.13 | -1.71E-02 | 2.81E-03 | 6.07E-10 | 6.78E-05 | 31 |
| 7 | 46398911 | rs17172691 | A | G | 0.09 | 1.84E-02 | 3.24E-03 | 7.10E-09 | 5.72E-05 | 26 |
| 7 | 46753491 | rs700750 | A | C | 0.63 | 1.29E-02 | 1.90E-03 | 7.42E-12 | 7.71E-05 | 35 |
| 7 | 73007943 | rs1051921 | A | G | 0.20 | -3.56E-02 | 2.29E-03 | 8.28E-55 | 4.03E-04 | 183 |
| 7 | 97822115 | rs11765552 | A | T | 0.54 | 1.54E-02 | 1.83E-03 | 2.20E-17 | 1.18E-04 | 54 |
| 7 | 128738482 | rs7795876 | T | C | 0.45 | 1.37E-02 | 1.87E-03 | 1.08E-13 | 9.34E-05 | 42 |
| 7 | 151413194 | rs10224210 | T | C | 0.72 | -2.67E-02 | 2.07E-03 | 2.38E-38 | 2.89E-04 | 131 |
| 7 | 155664686 | rs6971211 | T | C | 0.41 | 1.14E-02 | 2.01E-03 | 7.20E-09 | 6.26E-05 | 28 |
| 7 | 156254799 | rs7795670 | T | C | 0.80 | 1.58E-02 | 2.30E-03 | 4.00E-12 | 7.99E-05 | 36 |
| 8 | 23777006 | rs17786744 | A | G | 0.59 | -2.34E-02 | 1.85E-03 | 8.32E-37 | 2.65E-04 | 121 |
| 8 | 32461497 | rs10954864 | C | G | 0.49 | 1.39E-02 | 1.86E-03 | 3.34E-14 | 9.71E-05 | 44 |
| 8 | 38095662 | rs2306899 | T | C | 0.24 | -1.50E-02 | 2.15E-03 | 1.63E-12 | 8.27E-05 | 38 |
| 8 | 76478768 | rs2941484 | T | C | 0.45 | 3.22E-02 | 1.85E-03 | 1.82E-68 | 5.14E-04 | 233 |
| 8 | 95980708 | rs6982393 | T | C | 0.47 | -1.89E-02 | 1.84E-03 | 5.32E-25 | 1.78E-04 | 81 |
| 8 | 103642474 | rs2511661 | T | C | 0.14 | 1.97E-02 | 2.63E-03 | 3.93E-14 | 9.50E-05 | 43 |
| 8 | 130486168 | rs1835847 | T | C | 0.40 | 1.03E-02 | 1.90E-03 | 3.01E-08 | 5.10E-05 | 23 |
| 9 | 4118111 | rs6415788 | T | G | 0.60 | -1.07E-02 | 1.94E-03 | 1.58E-08 | 5.53E-05 | 25 |
| 9 | 20608912 | rs2188230 | T | C | 0.47 | 1.02E-02 | 1.86E-03 | 2.05E-08 | 5.21E-05 | 24 |
| 9 | 33084732 | rs12554192 | A | G | 0.73 | -1.37E-02 | 2.07E-03 | 2.12E-11 | 7.37E-05 | 33 |
| 9 | 33180362 | rs10813960 | T | C | 0.27 | -2.26E-02 | 2.09E-03 | 1.80E-27 | 2.03E-04 | 92 |
| 9 | 35686407 | rs1243872 | T | G | 0.54 | 1.06E-02 | 1.89E-03 | 1.01E-08 | 5.57E-05 | 25 |
| 9 | 71434707 | rs4744712 | A | C | 0.40 | -1.34E-02 | 1.88E-03 | 5.33E-13 | 8.58E-05 | 39 |
| 9 | 102090598 | rs323733 | A | T | 0.76 | -1.20E-02 | 2.18E-03 | 1.74E-08 | 5.31E-05 | 24 |
| 9 | 107690450 | rs1800977 | A | G | 0.33 | -1.41E-02 | 1.97E-03 | 3.99E-13 | 8.83E-05 | 40 |
| 9 | 130770276 | rs7874551 | T | G | 0.05 | -3.52E-02 | 4.48E-03 | 2.06E-15 | 1.06E-04 | 48 |
| 9 | 139105229 | rs3824359 | T | C | 0.86 | -1.90E-02 | 2.69E-03 | 8.72E-13 | 8.78E-05 | 40 |
| 10 | 16932196 | rs17343073 | A | T | 0.90 | -1.77E-02 | 3.07E-03 | 4.30E-09 | 5.79E-05 | 26 |
| 10 | 52643038 | rs17592117 | T | C | 0.87 | -4.21E-02 | 2.82E-03 | 9.34E-51 | 3.87E-04 | 176 |
| 10 | 52650502 | rs7918123 | A | G | 0.43 | 1.08E-02 | 1.89E-03 | 5.74E-09 | 5.72E-05 | 26 |
| 10 | 61469538 | rs1171614 | T | C | 0.23 | -5.57E-02 | 2.20E-03 | 1.18E-141 | 1.10E-03 | 502 |
| 10 | 69827012 | rs503677 | A | G | 0.35 | 1.26E-02 | 1.95E-03 | 5.62E-11 | 7.19E-05 | 33 |
| 10 | 82152638 | rs7916349 | A | G | 0.54 | -9.99E-03 | 1.87E-03 | 4.37E-08 | 4.97E-05 | 23 |
| 10 | 88939962 | rs7081609 | T | C | 0.15 | -2.29E-02 | 2.60E-03 | 7.24E-19 | 1.35E-04 | 61 |
| 10 | 104939232 | rs12240508 | A | G | 0.15 | 1.71E-02 | 2.57E-03 | 1.63E-11 | 7.42E-05 | 34 |
| 10 | 119388528 | rs181673 | A | C | 0.46 | -1.66E-02 | 1.84E-03 | 9.40E-20 | 1.36E-04 | 62 |
| 10 | 119480578 | rs10886117 | A | G | 0.17 | 1.85E-02 | 2.53E-03 | 1.29E-13 | 9.53E-05 | 43 |
| 10 | 119570121 | rs1925258 | A | T | 0.70 | -1.24E-02 | 2.03E-03 | 5.50E-10 | 6.51E-05 | 30 |
| 10 | 126419384 | rs10901812 | A | G | 0.27 | 1.71E-02 | 2.07E-03 | 9.18E-17 | 1.14E-04 | 52 |
| 10 | 134477720 | rs11146456 | A | G | 0.88 | -1.72E-02 | 2.96E-03 | 3.02E-09 | 6.07E-05 | 28 |
| 11 | 2121264 | rs7482894 | T | C | 0.65 | 1.47E-02 | 1.97E-03 | 4.14E-14 | 9.90E-05 | 45 |
| 11 | 2181395 | rs3842748 | C | G | 0.23 | 1.93E-02 | 2.27E-03 | 1.03E-17 | 1.32E-04 | 60 |
| 11 | 10236478 | rs7937758 | A | G | 0.50 | -1.24E-02 | 1.84E-03 | 8.22E-12 | 7.69E-05 | 35 |
| 11 | 30437981 | rs12806743 | T | G | 0.24 | -1.57E-02 | 2.24E-03 | 1.04E-12 | 8.93E-05 | 41 |
| 11 | 30749090 | rs963837 | T | C | 0.55 | 2.43E-02 | 1.84E-03 | 4.69E-40 | 2.92E-04 | 133 |
| 11 | 47410166 | rs11821917 | A | T | 0.14 | -1.47E-02 | 2.65E-03 | 1.66E-08 | 5.25E-05 | 24 |
| 11 | 61624885 | rs526126 | C | G | 0.81 | -1.63E-02 | 2.59E-03 | 1.70E-10 | 8.11E-05 | 37 |
| 11 | 64331462 | rs17300741 | A | G | 0.45 | 5.44E-02 | 1.86E-03 | 4.29E-188 | 1.47E-03 | 667 |
| 11 | 64520255 | rs7126110 | C | G | 0.86 | -5.16E-02 | 2.87E-03 | 1.26E-72 | 6.41E-04 | 291 |
| 11 | 65580638 | rs12576996 | T | G | 0.75 | -3.38E-02 | 2.12E-03 | 1.90E-57 | 4.24E-04 | 193 |
| 11 | 68860872 | rs11228490 | C | G | 0.33 | -1.07E-02 | 1.99E-03 | 3.48E-08 | 5.07E-05 | 23 |
| 11 | 75484929 | rs11823869 | C | G | 0.91 | -1.80E-02 | 3.29E-03 | 2.18E-08 | 5.05E-05 | 23 |
| 11 | 119235404 | rs2195525 | T | C | 0.52 | -1.63E-02 | 1.87E-03 | 1.89E-18 | 1.32E-04 | 60 |
| 11 | 120057343 | rs508205 | A | G | 0.56 | 1.31E-02 | 1.86E-03 | 8.16E-13 | 8.52E-05 | 39 |
| 12 | 15332342 | rs2193172 | C | G | 0.20 | -1.57E-02 | 2.35E-03 | 1.17E-11 | 7.99E-05 | 36 |
| 12 | 21329813 | rs11045819 | A | C | 0.16 | 1.47E-02 | 2.50E-03 | 2.32E-09 | 5.82E-05 | 26 |
| 12 | 50267335 | rs836968 | T | C | 0.27 | -1.56E-02 | 2.10E-03 | 5.11E-14 | 9.67E-05 | 44 |
| 12 | 52249665 | rs11169927 | A | G | 0.75 | 1.43E-02 | 2.13E-03 | 8.83E-12 | 7.75E-05 | 35 |
| 12 | 56865338 | rs2657879 | A | G | 0.82 | -2.10E-02 | 2.37E-03 | 3.69E-19 | 1.32E-04 | 60 |
| 12 | 57844049 | rs3741414 | T | C | 0.24 | -4.88E-02 | 2.16E-03 | 6.85E-113 | 8.76E-04 | 398 |
| 12 | 78773251 | rs12581220 | T | C | 0.32 | -1.39E-02 | 2.00E-03 | 1.73E-12 | 8.37E-05 | 38 |
| 12 | 96091058 | rs12423171 | A | G | 0.77 | -1.61E-02 | 2.19E-03 | 1.02E-13 | 9.20E-05 | 42 |
| 12 | 112007756 | rs653178 | T | C | 0.52 | -2.82E-02 | 1.82E-03 | 2.01E-54 | 3.98E-04 | 181 |
| 12 | 121436376 | rs1169303 | A | C | 0.51 | 1.26E-02 | 1.85E-03 | 4.54E-12 | 7.97E-05 | 36 |
| 12 | 122625992 | rs7953704 | A | G | 0.48 | -1.81E-02 | 1.84E-03 | 4.91E-23 | 1.63E-04 | 74 |
| 12 | 133069894 | rs12423664 | A | G | 0.14 | 2.28E-02 | 2.73E-03 | 2.87E-17 | 1.26E-04 | 57 |
| 13 | 31031884 | rs1360485 | T | C | 0.70 | 1.41E-02 | 2.02E-03 | 1.61E-12 | 8.36E-05 | 38 |
| 13 | 48640749 | rs2104480 | C | G | 0.29 | 1.39E-02 | 2.03E-03 | 3.28E-12 | 7.98E-05 | 36 |
| 13 | 72342605 | rs9572786 | A | G | 0.62 | 1.66E-02 | 1.90E-03 | 1.37E-18 | 1.29E-04 | 59 |
| 13 | 96130395 | rs2095774 | A | C | 0.10 | 2.00E-02 | 3.04E-03 | 2.26E-11 | 7.29E-05 | 33 |
| 13 | 97344738 | rs490016 | A | T | 0.79 | -1.33E-02 | 2.29E-03 | 2.94E-09 | 5.88E-05 | 27 |
| 14 | 37890433 | rs1955949 | T | C | 0.29 | 1.46E-02 | 2.03E-03 | 3.49E-13 | 8.72E-05 | 40 |
| 14 | 66110797 | rs11158607 | T | G | 0.34 | 1.18E-02 | 1.95E-03 | 6.59E-10 | 6.25E-05 | 28 |
| 14 | 73184100 | rs10149272 | C | G | 0.26 | -1.36E-02 | 2.15E-03 | 1.13E-10 | 7.07E-05 | 32 |
| 14 | 102686183 | rs7154553 | A | G | 0.80 | -1.76E-02 | 2.30E-03 | 9.82E-15 | 9.87E-05 | 45 |
| 14 | 103851272 | rs3759579 | A | G | 0.41 | -1.19E-02 | 1.87E-03 | 9.19E-11 | 6.91E-05 | 31 |
| 15 | 33342543 | rs345747 | A | G | 0.75 | 1.38E-02 | 2.13E-03 | 4.61E-11 | 7.09E-05 | 32 |
| 15 | 51106727 | rs3848132 | A | T | 0.29 | -1.23E-02 | 2.04E-03 | 7.76E-10 | 6.30E-05 | 29 |
| 15 | 63605954 | rs12708477 | A | C | 0.23 | 1.45E-02 | 2.22E-03 | 3.46E-11 | 7.35E-05 | 33 |
| 15 | 72112918 | rs12898868 | T | C | 0.25 | -1.78E-02 | 2.12E-03 | 2.53E-17 | 1.20E-04 | 54 |
| 15 | 74467796 | rs11854957 | T | C | 0.22 | -1.36E-02 | 2.28E-03 | 1.23E-09 | 6.33E-05 | 29 |
| 15 | 76298132 | rs4886755 | A | G | 0.49 | -2.92E-02 | 1.82E-03 | 3.65E-58 | 4.27E-04 | 194 |
| 15 | 81167779 | rs8025378 | T | C | 0.74 | -1.16E-02 | 2.13E-03 | 2.79E-08 | 5.17E-05 | 23 |
| 15 | 86013665 | rs17553790 | A | G | 0.58 | -1.29E-02 | 1.88E-03 | 3.20E-12 | 8.17E-05 | 37 |
| 15 | 90668979 | rs9672635 | T | C | 0.23 | 1.66E-02 | 2.20E-03 | 2.34E-14 | 9.72E-05 | 44 |
| 15 | 99287375 | rs12908437 | T | C | 0.36 | 3.23E-02 | 1.91E-03 | 2.37E-64 | 4.84E-04 | 220 |
| 16 | 3709523 | rs17183750 | A | G | 0.17 | 1.45E-02 | 2.49E-03 | 3.13E-09 | 5.99E-05 | 27 |
| 16 | 20366507 | rs13329952 | T | C | 0.81 | 1.56E-02 | 2.39E-03 | 3.14E-11 | 7.55E-05 | 34 |
| 16 | 21021657 | rs3103810 | A | C | 0.43 | 1.07E-02 | 1.86E-03 | 4.99E-09 | 5.59E-05 | 25 |
| 16 | 51735746 | rs9932625 | A | G | 0.23 | 1.72E-02 | 2.19E-03 | 2.02E-15 | 1.04E-04 | 47 |
| 16 | 53819169 | rs9936385 | T | C | 0.61 | -1.80E-02 | 1.89E-03 | 9.72E-22 | 1.54E-04 | 70 |
| 16 | 56481282 | rs4784669 | A | G | 0.41 | -1.01E-02 | 1.89E-03 | 4.07E-08 | 4.98E-05 | 23 |
| 16 | 68243486 | rs6499163 | T | G | 0.17 | -1.46E-02 | 2.43E-03 | 9.35E-10 | 6.12E-05 | 28 |
| 16 | 69552785 | rs4783581 | C | G | 0.77 | -2.76E-02 | 2.21E-03 | 5.37E-36 | 2.68E-04 | 122 |
| 16 | 79755446 | rs4575545 | A | G | 0.31 | -2.52E-02 | 2.00E-03 | 8.56E-37 | 2.71E-04 | 123 |
| 16 | 79843036 | rs6564708 | T | C | 0.12 | 1.63E-02 | 2.83E-03 | 3.75E-09 | 5.65E-05 | 26 |
| 16 | 79927218 | rs9935686 | A | C | 0.15 | 2.82E-02 | 2.57E-03 | 2.07E-28 | 2.00E-04 | 91 |
| 17 | 7760983 | rs3744251 | A | G | 0.08 | 2.20E-02 | 3.55E-03 | 2.60E-10 | 6.79E-05 | 31 |
| 17 | 19440538 | rs894680 | A | G | 0.39 | -1.09E-02 | 1.95E-03 | 1.12E-08 | 5.63E-05 | 26 |
| 17 | 21104817 | rs1109513 | A | C | 0.51 | 1.02E-02 | 1.86E-03 | 2.40E-08 | 5.15E-05 | 23 |
| 17 | 26955330 | rs12602520 | A | C | 0.91 | -1.79E-02 | 3.29E-03 | 2.49E-08 | 5.08E-05 | 23 |
| 17 | 42179281 | rs2523162 | A | G | 0.21 | 1.55E-02 | 2.25E-03 | 3.23E-12 | 7.99E-05 | 36 |
| 17 | 53364788 | rs7224610 | A | C | 0.60 | -2.84E-02 | 1.88E-03 | 4.77E-52 | 3.86E-04 | 175 |
| 17 | 59450105 | rs11657044 | T | C | 0.16 | -2.43E-02 | 2.53E-03 | 4.19E-22 | 1.63E-04 | 74 |
| 17 | 73343927 | rs9900586 | A | C | 0.21 | -1.22E-02 | 2.25E-03 | 2.49E-08 | 5.00E-05 | 23 |
| 17 | 74290566 | rs164106 | A | C | 0.39 | -1.33E-02 | 1.89E-03 | 8.56E-13 | 8.45E-05 | 38 |
| 18 | 5603310 | rs1719980 | A | G | 0.44 | -1.08E-02 | 1.92E-03 | 8.77E-09 | 5.77E-05 | 26 |
| 18 | 24337424 | rs527616 | C | G | 0.36 | 1.20E-02 | 2.04E-03 | 1.86E-09 | 6.67E-05 | 30 |
| 18 | 42777064 | rs10163551 | T | C | 0.30 | 1.29E-02 | 2.02E-03 | 7.77E-11 | 7.06E-05 | 32 |
| 18 | 43216010 | rs12959849 | T | C | 0.91 | -2.10E-02 | 3.25E-03 | 5.30E-11 | 7.40E-05 | 34 |
| 18 | 57850422 | rs538656 | T | G | 0.23 | 2.05E-02 | 2.17E-03 | 1.95E-21 | 1.50E-04 | 68 |
| 19 | 4995926 | rs11880464 | T | C | 0.23 | -1.34E-02 | 2.25E-03 | 1.41E-09 | 6.27E-05 | 28 |
| 19 | 7199978 | rs1035940 | C | G | 0.28 | 2.12E-02 | 2.04E-03 | 1.34E-25 | 1.81E-04 | 82 |
| 19 | 7226450 | rs11669336 | C | G | 0.68 | 1.52E-02 | 2.02E-03 | 2.00E-14 | 1.01E-04 | 46 |
| 19 | 18398628 | rs11086102 | C | G | 0.63 | -1.43E-02 | 1.91E-03 | 3.87E-14 | 9.47E-05 | 43 |
| 19 | 33364628 | rs8101881 | T | C | 0.61 | -1.61E-02 | 1.88E-03 | 4.64E-18 | 1.24E-04 | 56 |
| 19 | 45351746 | rs1871047 | A | G | 0.60 | -1.13E-02 | 1.92E-03 | 1.80E-09 | 6.16E-05 | 28 |
| 19 | 53462957 | rs7507133 | T | G | 0.33 | -1.31E-02 | 2.02E-03 | 5.27E-11 | 7.59E-05 | 34 |
| 20 | 10640201 | rs6040060 | A | G | 0.28 | 1.48E-02 | 2.21E-03 | 1.02E-11 | 8.83E-05 | 40 |
| 20 | 25257260 | rs2257370 | C | G | 0.18 | 1.55E-02 | 2.40E-03 | 4.87E-11 | 7.14E-05 | 32 |
| 20 | 33212055 | rs6142206 | A | G | 0.42 | 1.57E-02 | 1.86E-03 | 1.63E-17 | 1.20E-04 | 54 |
| 20 | 43042364 | rs1800961 | T | C | 0.03 | -5.13E-02 | 5.44E-03 | 2.19E-21 | 1.60E-04 | 73 |
| 20 | 43058018 | rs3746574 | T | C | 0.48 | 1.50E-02 | 1.89E-03 | 1.15E-15 | 1.12E-04 | 51 |
| 20 | 57472043 | rs2057291 | A | G | 0.35 | 1.07E-02 | 1.98E-03 | 3.61E-08 | 5.15E-05 | 23 |
| 20 | 62586262 | rs817318 | T | C | 0.60 | -1.07E-02 | 1.95E-03 | 2.23E-08 | 5.48E-05 | 25 |
| 21 | 16576783 | rs2823139 | A | G | 0.34 | 1.38E-02 | 1.95E-03 | 6.56E-13 | 8.52E-05 | 39 |
| 21 | 31587793 | rs686364 | A | G | 0.77 | -1.84E-02 | 2.18E-03 | 1.20E-17 | 1.22E-04 | 56 |
| 21 | 35356706 | rs2834317 | A | G | 0.15 | 1.75E-02 | 2.57E-03 | 4.60E-12 | 7.96E-05 | 36 |
| 21 | 37834641 | rs219778 | A | G | 0.74 | 2.09E-02 | 2.08E-03 | 4.06E-24 | 1.70E-04 | 77 |
| 22 | 30692353 | rs929454 | A | T | 0.20 | -1.48E-02 | 2.32E-03 | 9.22E-11 | 7.06E-05 | 32 |
| 22 | 32462564 | rs733907 | T | C | 0.92 | 1.99E-02 | 3.47E-03 | 4.88E-09 | 5.90E-05 | 27 |
| 22 | 44324727 | rs738409 | C | G | 0.78 | 1.87E-02 | 2.23E-03 | 2.71E-17 | 1.18E-04 | 53 |

## Table S3 – Multivariable Mendelian randomization estimates for the direct effect of serum urate and metabolic traits, on risk of coronary heart disease (CHD), peripheral arterial disease (PAD) and stroke.

| Exposure variable | Outcome | Direct effect (log OR) | Standard error | P |
| --- | --- | --- | --- | --- |
| Urate | CHD | 0.128 | 0.042 | 2.19E-03 |
| BMI | CHD | 0.272 | 0.047 | 9.45E-09 |
| eGFR | CHD | 0.137 | 0.349 | 3.92E-01 |
| T2DM | CHD | 0.114 | 0.019 | 3.15E-09 |
| LDL-C | CHD | 0.365 | 0.031 | <2E-16 |
| HDL-C | CHD | -0.025 | 0.038 | 5.19E-01 |
| TG | CHD | 0.076 | 0.042 | 7.10E-02 |
| Urate | PAD | 0.044 | 0.039 | 2.61E-01 |
| BMI | PAD | 0.337 | 0.044 | 3.85E-14 |
| eGFR | PAD | 0.164 | 0.320 | 6.10E-01 |
| T2DM | PAD | 0.179 | 0.018 | <2E-16 |
| LDL-C | PAD | 0.136 | 0.026 | 1.40E-07 |
| HDL-C | PAD | -0.053 | 0.035 | 1.31E-01 |
| TG | PAD | 0.096 | 0.039 | 1.44E-02 |
| Urate | Stroke | 0.105 | 0.032 | 1.09E-03 |
| BMI | Stroke | 0.110 | 0.036 | 2.55E-03 |
| eGFR | Stroke | -0.594 | 0.270 | 2.82E-02 |
| T2DM | Stroke | 0.071 | 0.015 | 1.45E-06 |
| LDL-C | Stroke | 0.094 | 0.023 | 4.89E-05 |
| HDL-C | Stroke | -0.025 | 0.029 | 3.92E-01 |
| TG | Stroke | -0.044 | 0.032 | 1.76E-01 |

*OR: odds ratio. Instruments and genetic associations with risk of body mass index BMI), estimated glomerular filtration rate (eGFR), type 2 diabetes mellitus (T2DM), low-density lipoprotein cholesterol (LDL-C), high-density lipoprotein cholesterol (HDL-C) and triglycerides (TG) are included together in the same regression model.*

## Table S4 – Multivariable Mendelian randomization estimates for the direct effect of 1-standard deviation increase in genetically determined serum urate and systolic blood pressure (SBP) respectively, on risk of coronary heart disease (CHD), peripheral arterial disease (PAD) and stroke.

| **Exposure** | **Outcome** | **OR** | **LCI** | **UCI** | **P** |
| --- | --- | --- | --- | --- | --- |
| Urate | CHD | 1.13 | 1.03 | 1.23 | 7.84E-03 |
| SBP | CHD | 1.84 | 1.56 | 2.15 | 8.91E-13 |
| Urate | PAD | 1.08 | 1.00 | 1.17 | 6.71E-02 |
| SBP | PAD | 1.76 | 1.52 | 2.04 | 3.03E-13 |
| Urate | Stroke | 1.06 | 0.99 | 1.12 | 9.79E-02 |
| SBP | Stroke | 1.76 | 1.57 | 1.98 | <2E-16 |

*LCI: lower 95% confidence interval; OR: odds ratio; UCI: upper 95% confidence interval.*

## Table S5 – Inverse-variance weighted Mendelian randomization analyses investigating the association of genetically proxied coronary heart disease (CHD), peripheral arterial disease (PAD), stroke and systolic blood pressure (SBP) on serum urate levels.

| **Exposure** | **Outcome** | **Variants** | **Estimate** | **Standard error** | **P** |
| --- | --- | --- | --- | --- | --- |
| CHD | Urate | 36 | 0.001 | 0.002 | 0.763 |
| PAD | Urate | 10 | 0.071 | 0.058 | 0.221 |
| Stroke | Urate | 15 | 0.068 | 0.042 | 0.104 |
| SBP | Urate | 70 | 0.002 | 0.007 | 0.763 |

*For binary exposures (CHD, PAD and stroke) units are in log odds ratios, and for SBP standard deviation units are used.*

## Table S6 – Characteristics of the UK Biobank populations considered and analyzed in one-sample Mendelian randomization.

|  | **Total, N** | **Female, N (% total)** | **Age, years (SD)** | **SBP, mmHg (SD)** | **Urate, µmol/L (SD)** |
| --- | --- | --- | --- | --- | --- |
| Considered population | 442,601 | 240,262 (54.3) | 56.9 (8.0) | 138.1  (18.6) | 309.3 (80.3) |
| Analyzed population | 392,282 | 211,752 (54.0) | 56.9 (8.0) | 138.1  (18.6) | 309.4 (80.3) |

*The analyzed population refers to that used when investigating systolic blood pressure (SBP) as an outcome. N: number; SD: standard deviation.*

## Table S7 – Missing data for the UK Biobank population considered in one-sample Mendelian randomization investigating systolic blood pressure as an outcome.

| **Variable** | **Number missing (% of total considered)** |
| --- | --- |
| Systolic blood pressure | 40,919 (9.2) |
| Principal components 1-4 of genetic ancestry | 11,568 (2.6) |
| Genetic risk score | 12,371 (2.8) |
| All | 50,319 (11.4) |

## Table S8 – Variants and their weights for the urate genetic risk score used in one-sample Mendelian randomization.

| Chromosome | Position (hg19) | SNP | Allele1 | Allele2 | Allele1 frequency | Weight |
| --- | --- | --- | --- | --- | --- | --- |
| 1 | 2156999 | rs2480712 | C | G | 0.6621 | 0.024325 |
| 1 | 15828704 | rs4646068 | T | C | 0.692 | 0.02366 |
| 1 | 27122152 | rs79598313 | T | C | 0.026 | 0.09962 |
| 1 | 145723120 | rs77923202 | A | T | 0.0197 | -0.17523 |
| 1 | 155157715 | rs10910845 | A | C | 0.4692 | 0.057964 |
| 1 | 163722075 | rs2070803 | A | G | 0.5777 | 0.052586 |
| 1 | 221038177 | rs139429135 | A | G | 0.515 | -0.03908 |
| 1 | 651349 | rs12037861 | A | T | 0.7041 | 0.023078 |
| 2 | 15788511 | rs2867112 | T | G | 0.8301 | 0.035023 |
| 2 | 27730940 | rs72782806 | A | G | 0.2595 | 0.025282 |
| 2 | 59321225 | rs1260326 | T | C | 0.3983 | 0.069602 |
| 2 | 69813458 | rs12472381 | A | G | 0.39 | 0.021931 |
| 2 | 121306440 | rs12987661 | T | C | 0.8657 | 0.041324 |
| 2 | 145509615 | rs17050272 | A | G | 0.421 | 0.031806 |
| 2 | 148844369 | rs373773367 | T | C | 0.0106 | 3.59331 |
| 2 | 170037294 | rs11683692 | T | C | 0.9444 | -0.04821 |
| 2 | 177278437 | rs1234413 | T | C | 0.4415 | -0.02235 |
| 2 | 211540507 | rs9287911 | A | T | 0.2497 | 0.038206 |
| 2 | 213083638 | rs187355703 | C | G | 0.9747 | -0.08614 |
| 2 | 53071797 | rs1047891 | A | C | 0.3107 | -0.02374 |
| 2 | 69145632 | rs9288447 | T | C | 0.5458 | -0.02253 |
| 3 | 125118082 | rs2581817 | C | G | 0.4198 | 0.047911 |
| 3 | 132288497 | rs11128111 | T | C | 0.48 | -0.02089 |
| 3 | 169155476 | rs7640441 | A | C | 0.2457 | -0.02753 |
| 3 | 8836576 | rs76651220 | A | C | 0.051 | 0.062033 |
| 3 | 9915325 | rs62294340 | A | G | 0.364 | -0.02186 |
| 4 | 10396709 | rs2368642 | A | G | 0.039 | -0.18286 |
| 4 | 48019323 | rs377261498 | A | T | 0.0398 | 0.118176 |
| 4 | 89039082 | rs191892884 | A | C | 0.9733 | 0.289179 |
| 4 | 89166761 | rs6825187 | T | C | 0.3573 | 0.187461 |
| 4 | 89208565 | rs10001622 | A | G | 0.4004 | -0.15072 |
| 4 | 144158285 | rs140268712 | T | G | 0.0187 | -0.09692 |
| 4 | 34660235 | rs98270 | A | G | 0.362 | 0.021718 |
| 4 | 72426137 | rs1481012 | A | G | 0.8893 | -0.24884 |
| 4 | 7214676 | rs7656569 | A | C | 0.1901 | -0.06735 |
| 4 | 25809716 | rs4693210 | A | G | 0.5571 | 0.030004 |
| 4 | 43386089 | rs1440411 | T | C | 0.5706 | -0.02755 |
| 4 | 43805502 | rs117467868 | A | G | 2.00E-04 | 5.88617 |
| 5 | 126223944 | rs455213 | T | C | 0.5428 | -0.02653 |
| 5 | 1286567 | rs10942549 | C | G | 0.312 | -0.04215 |
| 5 | 73017005 | rs677394 | T | G | 1.00E-04 | 5.74972 |
| 5 | 97845713 | rs76004499 | C | G | 0.9722 | -0.07367 |
| 6 | 151406005 | rs12530084 | T | C | 0.2198 | 0.06643 |
| 6 | 23748420 | rs1359232 | A | C | 0.466 | -0.09084 |
| 6 | 76479839 | rs2396025 | A | T | 0.2195 | 0.049872 |
| 6 | 95678312 | rs10223666 | C | G | 0.7036 | 0.046445 |
| 6 | 33125000 | rs4897160 | A | G | 0.4828 | 0.029735 |
| 7 | 130770484 | rs62435145 | T | G | 0.6891 | 0.041662 |
| 7 | 16920892 | rs77218295 | A | C | 1.00E-04 | 6.16973 |
| 7 | 52614189 | rs13226650 | A | G | 0.8085 | 0.048744 |
| 7 | 60242465 | rs11551890 | A | G | 0.5087 | 0.02308 |
| 7 | 61467182 | rs10480300 | T | C | 0.2755 | 0.030123 |
| 8 | 88880689 | rs34861762 | T | C | 0.4194 | 0.034282 |
| 8 | 114754784 | rs2943539 | T | C | 0.4754 | 0.04144 |
| 8 | 2165576 | rs10956924 | T | C | 0.2793 | -0.02387 |
| 8 | 30760335 | rs147532667 | T | C | 1.00E-04 | 5.1292 |
| 9 | 64332862 | rs10971420 | T | C | 0.6875 | 0.030745 |
| 9 | 64520255 | rs56106601 | A | C | 0.9455 | 0.060781 |
| 10 | 65580638 | rs74440730 | A | C | 0.8924 | -0.03682 |
| 10 | 119238381 | rs10994731 | A | G | 0.8816 | -0.06086 |
| 10 | 15359063 | rs11006159 | A | G | 0.2951 | -0.02383 |
| 10 | 52258777 | rs1171617 | T | G | 0.7682 | 0.079207 |
| 10 | 57751854 | rs9420446 | T | C | 0.1371 | -0.03802 |
| 10 | 111910219 | rs35198068 | T | C | 0.7063 | 0.024734 |
| 11 | 122500748 | rs35506085 | A | G | 0.1887 | -0.02876 |
| 11 | 133069894 | rs3925584 | T | C | 0.5522 | 0.030389 |
| 11 | 31029931 | rs71456318 | A | C | 0.4842 | 0.079027 |
| 11 | 72347696 | rs7126110 | C | G | 0.8554 | -0.0743 |
| 11 | 104167564 | rs12576996 | T | G | 0.7372 | -0.04687 |
| 11 | 39873321 | rs10892354 | T | C | 0.38 | 0.030101 |
| 12 | 73330423 | rs7303595 | A | T | 0.3357 | 0.025313 |
| 12 | 76304503 | rs11614136 | A | T | 0.6439 | -0.02906 |
| 12 | 99287375 | rs12313306 | T | C | 0.2464 | -0.07633 |
| 12 | 20365234 | rs10774625 | A | G | 0.4826 | 0.032335 |
| 12 | 53816275 | rs28530689 | A | C | 0.5117 | 0.032213 |
| 12 | 69575238 | rs12423664 | A | G | 0.1515 | 0.041927 |
| 13 | 72029069 | rs7986094 | A | C | 0.3016 | -0.02392 |
| 13 | 79753976 | rs626277 | A | C | 0.5938 | 0.025914 |
| 14 | 79927303 | rs149948615 | A | G | 0.9999 | -5.99874 |
| 14 | 19438321 | rs861536 | A | G | 0.621 | 0.0238 |
| 15 | 53356126 | rs9708153 | T | C | 0.0154 | 2.98553 |
| 15 | 59456589 | rs1478604 | T | C | 0.7061 | -0.02625 |
| 15 | 57876227 | rs6495044 | A | G | 0.7086 | 0.024401 |
| 15 | 4983304 | rs10851885 | A | G | 0.7559 | -0.05396 |
| 15 | 7211311 | rs12908437 | T | C | 0.3761 | 0.045764 |
| 16 | 18326222 | rs4997081 | C | G | 0.1962 | -0.03019 |
| 16 | 33350060 | rs8050136 | A | C | 0.4029 | 0.02464 |
| 16 | 49993535 | rs62052820 | A | G | 0.2124 | 0.041401 |
| 16 | 10643850 | rs12920245 | A | C | 0.1047 | 0.037576 |
| 16 | 33287782 | rs57652769 | T | C | 0.3094 | -0.0362 |
| 16 | 43038720 | rs9925837 | A | G | 0.8445 | -0.04163 |
| 17 | 37832621 | rs2453580 | T | C | 0.5977 | 0.024703 |
| 17 | 44325516 | rs4617927 | T | G | 0.446 | 0.035037 |
| 17 | 2156999 | rs9895661 | T | C | 0.8174 | 0.050208 |
| 18 | 15828704 | rs11663816 | T | C | 0.7295 | -0.03038 |
| 19 | 27122152 | rs117864137 | A | G | 0.731 | 0.028299 |
| 19 | 145723120 | rs10405423 | A | C | 0.6625 | 0.03865 |
| 19 | 155157715 | rs4808762 | T | C | 0.7199 | -0.02423 |
| 19 | 163722075 | rs2868194 | T | C | 0.4084 | -0.02676 |
| 19 | 221038177 | rs148062412 | A | T | 0.037 | 0.163684 |
| 19 | 651349 | rs62128132 | T | C | 0.9661 | -0.11756 |
| 20 | 15788511 | rs7267595 | A | C | 0.5097 | 0.022524 |
| 20 | 27730940 | rs6142206 | A | G | 0.4136 | 0.023316 |
| 20 | 59321225 | rs1800961 | T | C | 0.0339 | -0.07579 |
| 21 | 69813458 | rs219781 | T | G | 0.2456 | -0.02515 |
| 22 | 121306440 | rs4911647 | A | G | 0.9901 | -3.15857 |
| 22 | 145509615 | rs12485100 | T | G | 0.1726 | -0.03262 |

## Table S9 – Characteristics of studies identified in the updated systematic review.

| **Study (author, year)** | **Study Name (ref)** | **Year** | **Design** | **N**  **(total)** | **N (treatment)** | **N**  **(control)** | **Inclusion Criteria** | **Exclusion Criteria** | **Follow-up (days)** | **Primary Outcomes** | **Secondary Outcomes** | **Link to Public Trial Registry** |
| --- | --- | --- | --- | --- | --- | --- | --- | --- | --- | --- | --- | --- |
| Alshahawey 2017 | Effect of febuxostat on the endothelial dysfunction in hemodialysis patients: a randomized, placebo-controlled, double-blinded study (20) | 2017 | Parallel | 57 | 28 | 29 | Male or female. Maintenance haemodialysis. Age 18–70. Serum UA level ≥7.0 mg/dL. Stable clinical condition (no hospitalisation in the previous 3 months) | Current ULT. Hypersensitivity to febuxostat. Current treatment with mercaptopurine, azathioprine, pyrazinamide, or ethambutol. Participant in another clinical trial within the past four weeks. Judged to be unsuitable by the attending physician | 60 | Asymmetric Dimethylarginine physiological marker | Reduction in serum UA, reduction in serum high-sensitivity CRP, elevation of ALT or AST, occurrence of pancytopenia. | <https://clinicaltrials.gov/ct2/show/NCT02866214> |
| Dalbeth 2017 | Effects of febuxostat in early gout: a randomized, double-blind, placebo-controlled study (21) | 2017 | Parallel | 314 | 157 | 157 | Serum UA ≥7.0 mg/dL. Meet the American Rheumatology Association preliminary classification criteria for gout and have early gout (defined as having experienced ≤2 gout flares). Subjects with 2 gout flares could have experienced only 1 flare in the past 12 months. Eligible males were to be >18 years of age and eligible females were to be ≥45 years of age and >2 years postmenopausal or ≥55 years of age if receiving HRT. | Prior ULT. Secondary hyperuricemia. Rheumatoid arthritis. Active peptic ulcer disease. Xanthinuria. Myocardial infarction or stroke in 90 days prior to screening. Cancer not in remission for ≥5 years. eGFR of <60 mL/min/1.73m2. Serum creatinine of >2.0 mg/d., ALT/AST more than twice the upper limit of normal. History of drug or alcohol abuse in the past five years. Any issues that would prevent them from receiving an MRI scan. | 730 | Mean change joint erosion score | CFB to month 24 in the RAMRIS scores for erosion, oedema, and synovitis in the single affected joint. Incidence of gout flares. Serum uric acid levels | NCT010783 |
| Gingles 2019 | Allopurinol treatment adversely impacts left ventricular mass regression in patients with well-controlled hypertension (22) | 2019 | Parallel | 72 | 36 | 36 | Aged >18. Essential hypertension. Stable antihypertensive therapy for ≥3 months prior to study screening. Daytime average systolic BP <135mmHg. LVH based on American society of echocardiography criteria (males >115g/m2, females >95g/m2) | Intolerance to allopurinol. Left Ventricular Ejection Fraction <45%. Severe aortic stenosis. Active gout (flare within two years). Currently on allopurinol or azathioprine or 6-mercaptopurine or theophylline. Severe hepatic disease. CKD class 3B or worse. Malignancy (receiving active treatment) or other life-threatening diseases. Pregnant or lactating women. Any contraindication to MRI. Participation in any other clinical trial of an investigational medicinal product within the previous 30 days. Unable to give informed consent. Any other reason considered by a study physician to be inappropriate for inclusion. | 365 | The change in LV mass index with allopurinol versus placebo. | Percentage change in brachial artery diameter and change in augmentation index with allopurinol versus placebo. Change in average 24-hour BP control with allopurinol versus placebo. Change in CRP, BNP, troponin I, oxidized lactate dehydrogenase and procollagen carboxyl end peptide with allopurinol versus placebo. Change in LV mass, LV end systolic volume, LV end diastolic volume or LV ejection fraction. Change in LV mass after subtracting the volume of scar with allopurinol versus placebo | https://clinicaltrials.gov/ct2/show/NCT02237339 |
| Gunawhardana 2017 | Effect of febuxostat on ambulatory blood pressure in subjects with hyperuricemia and hypertension: a phase 2 randomized placebo-controlled study (23) | 2017 | Parallel | 121 | 61 | 60 | Hypertension - average clinic systolic BP of ≥145 mmHg and ≤165 mmHg or average clinic diastolic BP of ≥90 mmHg and ≤105 mmHg at the Day -21 Screening Visit; the average BP measurement at two of the three Placebo Run-in Visits (Day -14, Day -7 and Day -1) must also meet these criteria. Serum UA ≥7.0 mg/dL not associated with gout. 24-hour mean ambulatory systolic BP of ≥130 mmHg and <165 mmHg at the Baseline (Day 1) Visit. Maximum number of antihypertensive medications ≤2 (fixed-dose combination medications are considered 2 medications, including diuretics). Stable dose of this medication for ≥1 month prior to start of the initial Screening Visit (Day -21). Male aged ≥18. Female who is: surgically sterilized or postmenopausal. Ability to consent. In the opinion of the investigator, the participant is capable of understanding and complying with protocol requirements. | Received any investigational compound within 30 days, or within 5 half-lives of the compound (whichever is longer) prior to the Screening Visit. Received ULT in a previous clinical study or as a therapeutic agent. Gout diagnosis. Secondary hyperuricemia. Secondary hypertension of any aetiology. Myocardial infarction, heart failure, unstable angina, coronary artery bypass graft, or percutaneous coronary intervention within six months prior to screening. Irregular cardiac rhythm which leads to difficulty with interpretation of ambulatory blood pressure monitoring. Congestive heart failure, hypertensive encephalopathy, cerebrovascular accident, or transient ischemic attack. Type 1 or poorly controlled type 2 diabetes mellitus (HbA1c >8.0%) at Screening. History of infection with hepatitis B, hepatitis C, or human immunodeficiency virus. Average clinic systolic BP >165 mmHg or diastolic BP >105 mmHg at 1 or more visits during the Placebo Run-in Period. Average clinic systolic BP or diastolic BP measurement that increases or decreases by >10 mmHg between Placebo Run-in visits (Day -14 to Day -7, or Day -7 to Day -1, or Day -14 to Day -1). Immediate family member, study site employee, or in a dependent relationship with a study site employee who is involved in conduct of this study or may consent under duress. ALT) and/or AST values >2.0 times the upper limit of normal. Significant medical condition that would interfere with the treatment, safety or compliance with the protocol. Alcoholism or illicit drug abuse within five years prior to the Screening Visit or is currently consuming >14 alcoholic drinks per week. Known hypersensitivity or allergies to febuxostat or any components of the formulations of this compound. Taking or expected to take a medication as described in the excluded medication section. History of cancer that has not been in remission for at least five years prior to the first dose of study drug (does not apply to successfully resected basal cell or stage I squamous cell carcinoma of the skin). eGFR <30 mL/min/1.73m3 at the Day -21 Screening Visit. Non-compliant (<80% or >120%) with study medication during Placebo Run-In Period. Upper arm circumference <24 cm or >42 cm. Work shift includes any hour between 11 PM (2300) to 7 AM (0700). Baseline 24-hour ambulatory blood pressure reading of insufficient quality (as described in Appendix F of the protocol). | 42 | Change from baseline in 24-hour mean systolic BP measured by ambulatory blood pressure monitoring | Change from baseline in 24-hour mean diastolic BP measured by ambulatory blood pressure monitoring at week 6. Change from baseline in serum urate levels | https://clinicaltrials.gov/ct2/show/NCT01496469 |
| Huang 2017 | Clinical Study on efficacy of allopurinol in patients with acute coronary syndrome and its functional mechanism (24) | 2017 | Parallel | 100 | 50 | 50 | Not clearly defined. Acute coronary syndrome presentations June 2013-2014. | Patients involved in other clinical trials. Revascularisation surgery in last three months. Hepatic or renal insufficiency. Significant reduction in “blood cell counts”. Allergy to allopurinol | 730 | Not clearly defined. Indicators of oxidative stress and inflammatory response (malondialdehyde, oxidised low-density lipoprotein, nitric oxide, high sensitivity CRP and tumour necrosis factor alpha), as well as cardiovascular events during two year follow-up | Not defined | Not available |
| Kimura 2018 | Febuxostat therapy for patients with stage 3 CKD and asymptomatic hyperuricemia: a randomized trial (25) | 2018 | Parallel | 443 | 221 | 222 | Age ≥20. Hyperuricaemia (serum UA>7.0 and <10.0 mg/dL). eGFR >30 and <60 mL/min/1.73 m2. No history of gout. Obtained consent | Uncontrolled diabetes mellitus ((HbA1c ≥8.0% (JDS) or ≥8.4% (NGSP)). Systolic BP ≥160 mmHg or diastolic BP ≥100 mmHg. ALT or AST > two times the upper limit of normal. Change of serum creatinine >50% within 12 weeks. Acute renal disease, nephrotic syndrome, other serious disease, on dialysis, or renal-transplant. Complication or history of malignant tumour (not excluded if the malignant tumour is not treated within five years and if there is no recurrence). Hypersensitivity to febuxostat. Intake of any of the following drug at confirmation of eligibility; mercaptopurine hydrate, azathioprine, vidarabine, didanosine. Intake of ULT within four weeks before confirmation of eligibility. Initiation, discontinuation or dose change of any of the following drug within four weeks before confirmation of eligibility; losartan, fenofibrate, thiazide diuretics, loop diuretic. Continuous intake of salicylic acid drugs (not excluded if taking low-dose aspirin [324 mg/day]). HRT. Pregnancy, nursing or planning to become pregnant during the study. Participation in other clinical trials within 24 weeks before informed consent. Judged as ineligible in the opinion of the investigator | 756 | eGFR slope (change per year, mL/min/1.73 m2/year) | Changes of eGFR from the baseline to 108 weeks: change (mL/min/1.73 m2) and the rate of change (%) of eGFR from the baseline to 24, 48, 72, and 108 weeks. Changes of the serum uric acid from the baseline to 108 weeks: change (mg/dL) and the rate of change (%) of serum uric acid from the baseline to 108 weeks. Achievement of serum uric acid level <6.0 mg/dL. Occurrence of events indicating the deterioration of renal functions: Induction of dialysis and evaluation of the doubling of serum creatinine level. Changes of various markers from the baseline to 108 weeks: renal function (serum cystatin C), oxidative stress (urinary 8-OHdG, urinary L-FABP), inflammation (serum CRP), cardiovascular events (12-lead electrocardiogram, serum NT-pro-BNP, the albumin urine / creatinine ratio). Incidence of gouty arthritis. Incidence of adverse event | https://upload.umin.ac.jp/cgi-open-bin/ctr/ctr.cgi?function=brows&action=brows&recptno=R000009817&type=summary&language=E |
| McMullan 2017 | Effect of uric acid lowering on renin-angiotensin-system activation and ambulatory BP: a randomized controlled trial (26) | 2017 | Parallel | 149 | 47 Probenacid,  49 Allopurinol | 53 | 25 hydroxyvitamin D <20 ng/mL OR Uric acid ≥ 5 mg/dL. Age ≥18 and ≤75. Body Mass Index ≥25 kg/m^2 | Hypertension, or on BP-lowering medicine. Diabetes. Coronary Heart Disease. eGFR <60 mL/min. Kidney stones. Active cancer (except non-melanoma skin cancer). Pregnant. Taking vitamin D supplements and unwilling to stop. Osteoporosis. Hypo- or hypercalcemia. Hypo- or hyperphosphatemia. Known allergy to ACE-inhibitors. Taking medication for hyperuricemia. Gout. Anaemia. Cirrhosis. Active/chronic hepatitis. Abnormal AST, ALT or total bilirubin levels. Known allergy to either allopurinol or probenecid. Current use of didanosine, azothioprine, methotrexate, ketoprofen, ketorolac, mycophenolate, or ACE-inhibitors | 56 | Change in renal plasma flow response to captopril in high sodium balance. Plasma renin activity. Angiotensin II concentration | Change in Endothelium-Dependent Vasodilation. Mean 24-Hour Ambulatory BP. Mean 24-Hour Ambulatory BP. Nocturnal Dipping | https://clinicaltrials.gov/ct2/show/NCT01320722 |
| Morgan 2018 | Effects of losartan and allopurinol on cardiorespiratory regulation in obstructive sleep apnoea (27) | 2018 | Parallel | 58 | 30 | 28 | Sleep apnoea (apnoea-hypopnoea index ≥25 events / hour)  Clinical diagnosis of hypertension or two BP readings >140/90 mmHg in past 12 months | Cardiovascular disease other than hypertension, pulmonary disease with hypoxemia, hypertriglyceridemia, diabetes, kidney disease, hyperkalaemia, previous adverse reaction to allopurinol or losartan, patients receiving angiotensin converting enzyme inhibitors, alpha-adrenergic and angiotensin receptor antagonists, potassium-sparing diuretics without accompanying loop/thiazide diuretics, allopurinol, oxypurinol, febuxostat, amoxicillin, ampicillin, azathioprine, or mercaptopurine. | 42 | Muscle sympathetic nerve activity (MSNA) response to acute hypoxic exposure | Hyperoxic inhibition of MSNA, and ventilatory and vasodilatory responses to hypoxia | https://clinicaltrials.gov/ct2/show/NCT01637623 |
| Mukri 2018 | Role of febuxostat in retarding progression of diabetic kidney disease with asymptomatic hyperuricemia: A 6-months open-label, randomized controlled trial (28) | 2018 | Parallel | 100 | 50 | 50 | Age ≥18 and ≤75. CKD (eGFR range 15 to 60 ml/min/1.72 m2). Good glycaemic control of diabetes mellitus (HbA1c < 8.0 %). Asymptomatic hyperuricemia with serum UA ≥400 μmol/L. Never on any ULT. Optimal tolerated dose of anti-proteinuric agents. | Renal replacement therapy. Allergy to febuxostat. Heart failure. Gouty arthritis. Chronic liver disease of all aetiologies. | 182 | eGFR decline in CKD3/4 | Proteinuria. Adverse effects including cardiovascular events i.e. myocardial infarction, stroke, heart failure and death. | Not available |
| Omrani 2016 | The effect of allopurinol treatment regimen on serum uric acid and arterial blood pressure in haemodialysis patients (29) | 2016 | Parallel | 146 | 73 | 73 | Haemodialysis patients (≥3-month history). No ULT. No use of diuretic. | Lack of regular referral of the patient. Severe side-effects leading to termination of drug consumption | 90 | Effects of allopurinol on serum uric acid and BP in haemodialysis patients | Not defined | Not available |
| Saag 2019 | Efficacy and safety of febuxostat extended and immediate release in patients with gout and renal impairment: a phase III placebo-controlled study (30) | 2019 | Parallel | 1790 | 1426 (4 groups) | 357 | Capable of understanding and complying with protocol requirements and consent. History or presence of gout defined as having one or more of the American Rheumatism Association (ARA) criteria for the diagnosis of gout. Male or female aged ≥18. A female participant of childbearing potential agrees to use routinely adequate contraception. Serum urate UA ≥8.0 mg/dL at the Day -4 Visit or at the retest visit. eGFR ≥30 mL/min and <60 mL/min at Screening visit (Day -21 for participants on urate lowering therapy (ULT) and Day -4 for participants not on ULT) or at the retest visit. At least one gout flare within 12 months prior to Screening visit. | Received any investigational compound within 30 days prior to Screening. Immediate family member, study site employee, or is in a dependant relationship with a study site employee who is involved in conduct of this study or may consent under duress. Breastfeeding or pregnant. Secondary hyperuricemia. History of xanthinuria. Has received ULT within 20 days prior to Day 1/Randomization Visit. Hypersensitivity to febuxostat, aspirin, lansoprazole, colchicine or non-steroidal anti-inflammatory drug or any components of their formulation. Active peptic ulcer disease. History of cancer (other than basal cell carcinoma of the skin) within 5 years prior to the Screening Visit. ALT and AST >2 times the upper limit of normal. Rheumatoid arthritis which requires treatment. Significant medical condition and/or conditions that would interfere with the treatment, safety, or compliance with the protocol. Myocardial infarction (MI), stroke, hospitalized unstable angina, cardiac or cerebrovascular revascularization procedure or hospitalized transient ischemic attack (TIA). Drug abuse (defined as any illicit drug use) or alcohol abuse (>14 alcoholic beverages/week) within 5 years prior to the Screening visit. Participation in another investigational study within the 30 days prior to the Screening Visit. History of infection with hepatitis B, hepatitis C, or human immunodeficiency virus. Required to take excluded medications. | 90 | Percentage of participants with serum urate <5.0 mg/dl | Percentage of participants with at least one gout flare requiring treatment. Percentage of participants with serum urate <6.0 mg/dl | https://clinicaltrials.gov/ct2/show/NCT02139046 |
| Tausche 2017 | Lesinurad monotherapy in gout patients intolerant to a xanthine oxidase inhibitor: a 6-month phase III clinical trial and extension study (31) | 2017 | Parallel | 214 | 107 | 107 | Ability to consent. Meets the diagnosis of gout as per the American Rheumatism Association Criteria for the Classification of Acute Arthritis of Primary Gout. Serum UA level ≥ 6.5 mg/dL at the Screening and Day -7 Visits. Able to take gout flare prophylaxis with colchicine or NSAID (including Cox-2 selective NSAID) ± PPI. History of intolerance or a contraindication to either allopurinol or febuxostat. Body mass index <45 kg/m2 | Taking any other approved ULT at the Screening Visit. Documented history or suspicion of kidney stones. Pregnant or breastfeeding. Alcohol abuse. History or suspicion of drug abuse within the past 5 years. Requires or may require systemic immunosuppressive or immunomodulatory treatment. Known or suspected human immunodeficiency virus (HIV) infection. Positive test for active hepatitis B or hepatitis C infection. History of malignancy within the previous 5 years with the exception of non-melanoma skin cancer that has been treated with no evidence of recurrence, treated cervical dysplasia or treated in situ Grade 1 cervical cancer. Within the last 12 months with: unstable angina, New York Heart Association class III or IV heart failure, myocardial infarction, stroke, or deep venous thrombosis; or subjects currently receiving anticoagulants. Uncontrolled hypertension. Estimated creatinine clearance < 30 mL/min. Active peptic ulcer disease requiring treatment. Active liver disease or hepatic dysfunction. Chronic treatment with more than 325mg salicylates per day. taking valpromide, progabide, or valproic acid. Received an investigational therapy within 8 weeks or 5 half-lives (whichever is longer) prior to the Screening Visit. Any other medical or psychological condition, which in the opinion of the Investigator and/or Medical Monitor, might create undue risk to the subject or interfere with the subject's ability to comply with the protocol requirements, or to complete the study. | 196 | Number of subjects with a serum uric acid level that is < 6.0 mg/dL | Not defined | https://clinicaltrials.gov/ct2/show/NCT01508702 |
| Wada 2018 | Uric acid-lowering and renoprotective effects of topiroxostat, a selective xanthine oxidoreductase inhibitor, in patients with diabetic nephropathy and hyperuricemia: a randomized, double-blind, placebo-controlled, parallel-group study (UPWARD study) (32) | 2018 | Parallel | 65 | 43 | 22 | Age 20–75 years. Diabetes receiving drug treatment. Constant diet and/or exercise therapy for more than 8 weeks before the start of the pre-observation period. Clinically or pathologically diagnosed diabetic kidney disease. Gout or hyperuricemia. Three measurements of first-morning-void UACR in the observation period (Visit 1 or 2), more than two of which were ≥ 45 and < 300 mg/gCr. eGFR ≥ 30 mL/min/1.73 m2 at Visit 1. Outpatient (having no plan of hospital admission). Ability to consent. | Renal disease other than diabetic nephropathy (excluding nephrosclerosis). Received steroid preparations and immunosuppressants for the purpose of treating nephropathy within the past 10 years. Systemic diseases other than diabetes that induce proteinuria. Serum creatinine level that changed by 0.5 mg/dL or more at Visit 1 and 2. Serum UA level was 10.0 mg/dL or more at Visit 1. Nephrectomy or renal transplantation. Acute renal injury within 24 weeks before the start of the pre-observation period. Hepatic dysfunction. Serious heart disease. Serious hematologic disease. Cancer. Uncontrolled hypertension. Uncontrolled diabetes. Uncontrolled dyslipidaemia. Pregnancy. Breastfeeding. Onset of gouty arthritis at the start of the observation period. Using ULT, agents potentially affecting the SUA level and/or agents that could potentially cause adverse drug interactions with the study drug for at least 2 weeks before Visit 1. Judged to be clinically inappropriate by the investigators or sub-investigators. | 196 | Change in first void urinary ACR | Changes in UACR in spot urine, eGFR and serum UA levels | Not available |

*ACE – angiotensin converting enzyme; ALT – alanine aminotransferase; AST – aspartate aminotransferase; BP – blood pressure; CFB – change from baseline; CKD – chronic kidney disease; CRP – C-reactive protein; dL – decilitre; eGFR – estimated glomerular filtration rate - calculated using the Modification of Diet in Renal Disease (MDRD) equation; HBA1c – glycosylated haemoglobin; HRT – hormone-replacement therapy; JDS – Japan Diabetes Society; LV – left ventricle; LVH – left ventricular hypertrophy; mg – milligrams; mL – millilitre; mmHg – millimetres of mercury; MRI – magnetic resonance imaging; ng – nanograms; NGSP – National Glycohaemoglobin Standardisation Programme; NSAID – non-steroidal anti-inflammatory drug; NT-pro-BNP – NT-proB-type natriuretic peptide; RAMRIS – rheumatoid arthritis magnetic resonance imaging score; UA – uric acid; UACR – urinary albumin-creatinine ratio; ULT – urate-lowering therapy; Umol – micromole*

## Table S10 – Risk of bias for studies included in the systematic review and meta-analysis.

| Study/Year | **OUTCOME(S)** | **RANDOM SEQUENCE GENERATION** | **ALLOCATION CONCEALMENT** | **BLINDING OF PARTICIPANTS AND PERSONNEL** | **BLINDING OF OUTCOME ASSESSMENT** | **INCOMPLETE OUTCOME DATA** | **SELECTIVE REPORTING** | **OTHER BIAS** | **OVERALL RISK OF BIAS** |
| --- | --- | --- | --- | --- | --- | --- | --- | --- | --- |
| Akhondzadeh 2005 | MACE | L | U | L | L | L | L | L | U |
| Akhondzadeh 2006 | MACE | U | U | U | U | L | L | L | U |
| Alshahawey 2017 | SBP | L | L | U | U | L | U | L | U |
| Becker 2005 | MACE | U | U | U | U | L | L | L | U |
| Borgi 2017 | MACE | L | L | L | L | L | L | L | L |
| Bowden 2013 | MACE | U | U | U | U | L | U | L | U |
| Brunstein 2005 | MACE | U | U | U | U | L | L | L | U |
| Chen 2009 | MACE | U | U | H | H | L | U | U | H |
| Cingolani 2006 | MACE | U | U | L | L | L | L | L | U |
| Dalbeth 2017 | MACE | U | U | U | U | U | L | U | U |
| Dawson 2009 | MACE, MACE AND PRIOR CVD | L | L | L | L | U | L | U | U |
| Deng 2010 | MACE | U | U | H | H | U | U | H | H |
| Dickerson 2009 | MACE | U | U | U | U | L | L | L | U |
| Dogan 2011 | MACE, SBP | U | U | H | H | L | U | L | H |
| Fan 2012 | MACE | U | U | U | U | L | U | L | U |
| Feuerman 1973 | MACE | U | U | L | L | L | U | U | U |
| George 2006 | MACE | U | U | U | U | L | U | L | U |
| Gibson 1982 | MACE | U | U | H | H | L | H | U | H |
| Gingles, 2019 | SBP | L | U | U | H | U | L | L | H |
| Givertz 2015 | MACE | L | L | L | L | L | L | L | L |
| Goicoechea 2010 | SBP | L | U | H | H | L | L | L | H |
| Goldfarb 2013 | MACE | U | U | L | L | L | L | L | U |
| Greig 2011 | MACE | L | U | U | U | L | L | L | U |
| Gunawhardana 2017 | MACE, SBP | L | U | U | U | L | U | L | U |
| Hare 2008 | MACE | U | U | U | U | L | L | L | U |
| Higgins 2014 | MACE, MACE AND PRIOR CVD, SBP | L | L | L | L | L | L | L | L |
| Hill 2013 | MACE | L | L | L | L | L | L | L | L |
| Hosoya 2014 | MACE | U | U | U | U | L | L | L | U |
| Hosoya 2016 phase 2a | MACE | L | L | L | L | L | L | L | L |
| Hosoya 2016 phase 2b | MACE | L | L | L | L | L | L | L | L |
| Huang, 2017 | MACE, MACE AND PRIOR CVD | H | H | H | H | H | H | U | H |
| Jahangard 2014 | MACE | L | L | L | L | L | U | L | U |
| Jalal 2016 | MACE | L | L | U | U | L | L | L | U |
| Jalalzadeh 2012 | MACE | U | U | H | H | U | U | L | H |
| Jarnerot 2000 | MACE | U | U | U | U | L | L | L | U |
| Jitapunkul 1991 | MACE | U | U | U | U | L | U | L | U |
| Joelsson 2001 | MACE | U | U | U | U | L | U | L | U |
| Kamatani 2011 late phase 2 | MACE | L | L | U | U | L | L | L | U |
| Kamatani 2011 phase 3 | MACE | L | L | L | L | L | L | L | L |
| Kanbay 2011 | MACE, SBP | L | U | H | H | L | U | L | H |
| Khan 2008 | MACE, MACE AND PRIOR CVD, SBP | U | U | L | L | L | L | L | U |
| Kimura 2018 | SBP | L | L | L | L | U | L | U | U |
| Lei 2009 | MACE | U | U | H | H | H | L | U | H |
| Liu 2007 | MACE | U | U | H | H | U | U | H | H |
| Liu 2015 | MACE | L | U | H | H | H | L | L | H |
| Machado Vieira 2008 | MACE | U | U | L | L | L | L | L | U |
| Madero 2015 | MACE | L | L | L | L | L | L | L | L |
| Mao 2015 | MACE | L | U | U | U | U | U | U | U |
| McMullan 2017 | SBP | L | L | L | U | L | L | L | U |
| Modabber 2009 | MACE | U | U | U | U | L | L | L | U |
| Momeni 2010 | MACE, SBP | U | U | U | U | L | L | L | U |
| Morgan 2018 | SBP | L | U | L | L | U | U | H | U |
| Muir 2008 | MACE, MACE AND PRIOR CVD | L | L | L | L | L | L | U | U |
| Mukri 2018 | MACE | L | H | H | H | U | U | H | H |
| NCT01350388 2016 | MACE | U | U | L | L | L | L | L | U |
| NCT02128490/Gunawardhana 2016 | MACE | U | U | L | L | L | L | L | U |
| Noman 2010 | MACE | L | L | L | L | L | L | L | L |
| Omrani, 2016 | SBP | U | U | U | U | U | H | H | H |
| Poiley 2016 | MACE | U | U | L | L | L | L | L | U |
| Puntoni 2013 | MACE | L | L | L | L | L | L | L | L |
| Rassi 2007 | MACE | L | L | L | L | L | L | L | L |
| Rekhraj 2013 | MACE | L | U | U | L | L | U | L | U |
| Rentoukas 2010 | MACE, MACE AND PRIOR CVD | U | U | U | U | L | L | L | U |
| Robertson 2015 | MACE | L | L | L | L | L | L | L | L |
| Saag 2016 | MACE | U | U | L | L | L | L | U | U |
| Saag 2019 | MACE | L | U | U | U | H | L | U | H |
| Sarris 2007 | MACE | U | U | H | H | L | H | U | H |
| Schumacher 2008 | MACE | U | U | L | L | L | L | L | U |
| Segal 2015 | MACE, SBP | U | U | L | L | L | L | L | U |
| Separham 2016 | MACE, MACE AND PRIOR CVD | L | U | U | L | L | L | L | U |
| Shen 2010 | MACE | U | U | H | H | U | U | H | H |
| Shi 2012 | MACE | L | L | H | H | L | L | L | H |
| Sircar 2015 | MACE | L | L | L | L | L | L | L | L |
| Siu 2006 | MACE, SBP | L | U | H | H | L | U | L | H |
| Szwejkowski 2013 | MACE | L | U | L | L | L | U | L | U |
| Taheraghdam 2014 | MACE, MACE AND PRIOR CVD | L | L | L | L | L | L | L | L |
| Takir 2015 | MACE | U | H | H | H | L | U | U | H |
| Tanaka 2015 | MACE | L | L | H | H | L | U | L | H |
| Tani 2015 | MACE | U | L | H | H | L | L | L | H |
| Tausche, 2017 | MACE | U | U | U | U | U | U | L | U |
| Togha 2007 | MACE | L | L | L | L | U | L | U | U |
| Tsuruta 2015 | MACE | L | U | H | H | L | L | L | H |
| Usharani 2016 | MACE | L | U | U | U | L | L | L | U |
| Wada 2018 | MACE | U | U | U | U | U | U | U | U |
| Wang 2012 | MACE | L | U | H | H | U | L | U | H |
| Wang 2015 | MACE | L | U | H | H | L | U | U | H |
| Weiser 2012 | MACE | L | L | L | L | L | L | L | L |
| Weiser 2014 | MACE | L | L | L | L | L | L | L | L |
| Yin 2015 | MACE, MACE AND PRIOR CVD | U | U | H | H | L | L | U | H |
| Zhang 2012 | MACE, MACE AND PRIOR CVD | U | U | H | H | L | L | U | H |
| Zhou 2009 | MACE | U | U | H | H | U | H | H | H |
| Ziaee 2006 | MACE | U | U | U | U | L | U | L | U |

*H: high; L: low; U: undetermined; MACE: major adverse cardiovascular events; SBP: systolic blood pressure; CVD: cardiovascular disease*

## Table S11 – Baseline, absolute change and relative change in serum urate concentration in the included clinical trials.

| Study/Year | **OUTCOME(S)** | **Serum Urate Concentration Entry Criteria (mg/dL)** | **Baseline serum urate concentration**  **(mg/dL)** | | **Absolute change in serum**  **urate concentration**  **(Mean, mg/dL)** | **Proportional mean change in serum urate concentration (mg/dL)** |
| --- | --- | --- | --- | --- | --- | --- |
|  |  |  | **Mean** | **Standard Deviation** |  |  |
| Akhondzadeh 2005 | MACE | NA | - | - | - | - |
| Akhondzadeh 2006 | MACE | - | - | - | - | - |
| Alshahawey 2017 | SBP | ≥7.0 | 7.5 | 0.7 | -1.23 | -0.16 |
| Becker 2005 | MACE | ≥8.0 | 9.66 | 1.2 | - | - |
| Borgi 2017 | MACE | ≥5.0 | 6.1 | 0.9 | -1.78 | -0.29 |
| Bowden 2013 | MACE | - | 8.3 | 2.68 | -0.75 | -0.09 |
| Brunstein 2005 | MACE | - | 4.69 | 1.84 | - | - |
| Chen 2009 | MACE | NA | - | - | - | - |
| Cingolani 2006 | MACE | - | 7.62 | 0.15 | -7.62 | -1.00 |
| Dalbeth 2017 | MACE | ≥7.0 | 8.75 | 1.36 | -1.80 | -0.21 |
| Dawson 2009 | MACE, MACE AND PRIOR CVD | - | 5.72 | 1.59 | -0.84 | -0.15 |
| Deng 2010 | MACE | NA | - | - | - | - |
| Dickerson 2009 | MACE | - | - | - | - | - |
| Dogan 2011 | MACE, SBP | - | 5.15 | 1.59 | -1.20 | -0.23 |
| Fan 2012 | MACE | "Normal range" | - | - | - | - |
| Feuerman 1973 | MACE | - | - | - | - | - |
| George 2006 | MACE | - | 7.12 | 1.53 | - | - |
| Gibson 1982 | MACE | - | 6.52 | 1.08 | -0.91 | -0.14 |
| Gingles, 2019 | SBP | - | 6.09 | 1.63 | -1.61 | -0.26 |
| Givertz 2015 | MACE | ≥9.5 | 11.1 | 8.12 | -2.68 | -0.24 |
| Goicoechea 2010 | SBP | - | 7.55 | 1.88 | -0.81 | -0.11 |
| Goldfarb 2013 | MACE | - | 6.27 | 1.52 | - | - |
| Greig 2011 | MACE | - | 7.29 | 0.41 | -1.47 | -0.20 |
| Gunawhardana 2017 | MACE, SBP | - | - | - | -1.52 | - |
| Hare 2008 | MACE | - | 7.92 | 2.35 | - | - |
| Higgins 2014 | MACE, MACE AND PRIOR CVD, SBP | - | 5.21 | 1.51 | -0.84 | -0.16 |
| Hill 2013 | MACE | NA | - | - | - | - |
| Hosoya 2014 | MACE | >8 or >7 with gout | 8.5 | 1.2 | -2.27 | -0.27 |
| Hosoya 2016 phase 2a | MACE | >8 | 9.2 | 1 | -2.00 | -0.22 |
| Hosoya 2016 phase 2b | MACE | ≥9 or ≥7 with gout | 9.1 | 1.3 | -2.83 | -0.31 |
| Huang, 2017 | MACE, MACE AND PRIOR CVD | - | 9.82 | 0.58 | -2.35 | -0.24 |
| Jahangard 2014 | MACE | - | 5.2 | 1.3 | -1.55 | -0.30 |
| Jalal 2016 | MACE | >7.0 (male) >6.0 (female) | 8.5 | 1.5 | -1.55 | -0.18 |
| Jalalzadeh 2012 | MACE | > 6.5 (male)  > 5.5  (female) | 7.71 | 1.53 | -2.50 | -0.32 |
| Jarnerot 2000 | MACE | - | - | - | - | - |
| Jitapunkul 1991 | MACE | NA | - | - | - | - |
| Joelsson 2001 | MACE | - | - | - | - | - |
| Kamatani 2011 late phase 2 | MACE | NA | - | - | - | - |
| Kamatani 2011 phase 3 | MACE | >8 | 8.87 | 0.81 | -2.33 | -0.26 |
| Kanbay 2011 | MACE, SBP | >7 | 8.08 | 0.92 | -1.50 | -0.19 |
| Khan 2008 | MACE, MACE AND PRIOR CVD, SBP | ≥6.39 | 7.82 | 1.12 | -1.85 | -0.24 |
| Kimura 2018 | SBP | >7-10 | 7.8 | 0.9 | - | - |
| Lei 2009 | MACE | NA | - | - | - | - |
| Liu 2007 | MACE | NA | - | - | - | - |
| Liu 2015 | MACE | 7-8 | 7.3 | 0.2 | -0.75 | -0.10 |
| Machado Vieira 2008 | MACE | - | 4.7 | 0.2 | -0.89 | -0.19 |
| Madero 2015 | MACE | NA | - | - | - | - |
| Mao 2015 | MACE | NA | - | - | - | - |
| McMullan 2017 | SBP | ≥5.0 | 6.1 | 0.9 | -1.66 | -0.27 |
| Modabber 2009 | MACE | - | 4.5 | 0.5 | - | - |
| Momeni 2010 | MACE, SBP | - | 6.23 | 1.78 | -0.36 | -0.06 |
| Morgan 2018 | SBP | - | - | - | -0.92 | - |
| Muir 2008 | MACE, MACE AND PRIOR CVD | - | 5.76 |  | -0.81 | -0.14 |
| Mukri 2018 | MACE | ≥6.72 | 9.09 | 1.47 | -1.80 | -0.20 |
| NCT01350388 2016 | MACE | ≥4.60 | 7.16 | 1.4 | -1.58 | -0.22 |
| NCT02128490/Gunawardhana 2016 | MACE | ≥8.0 | 9.6 | 1.28 | - | - |
| Noman 2010 | MACE | - | 6.05 | 1 | -1.73 | -0.29 |
| Poiley 2016 | MACE | 7.5-12 | 9.1 | 1.5 | -1.69 | -0.19 |
| Puntoni 2013 | MACE | - | - | - | - | - |
| Rassi 2007 | MACE | - | - | - | - | - |
| Rekhraj 2013 | MACE | - | 9.66 | 1.96 | - | - |
| Rentoukas 2010 | MACE, MACE AND PRIOR CVD | - | 6.95 | 1.39 | - | - |
| Robertson 2015 | MACE | - | 5.89 | 1.49 | -1.86 | -0.32 |
| Saag 2016 | MACE | >7.0 | 10.5 | 1.7 | - | - |
| Saag 2019 | MACE | ≥8.0 | 9.61 | 1.27 | - | - |
| Sarris 2007 | MACE | >7.0 | 9.02 | 1.35 | -1.35 | -0.15 |
| Schumacher 2008 | MACE | ≥8.0 | 9.85 | 1.26 | - | - |
| Segal 2015 | MACE, SBP | - | 6.7 | 1.56 | -1.19 | -0.18 |
| Separham 2016 | MACE, MACE AND PRIOR CVD | - | 5.2 | 1.7 | - | - |
| Shen 2010 | MACE | >7  (male) >5.88 (female) | 8.95 | 1.43 | - | - |
| Shi 2012 | MACE | >7  (male)  > 6  (female) | 7.85 | 1.09 | -1.35 | -0.17 |
| Sircar 2015 | MACE | ≥7 | 8.59 | 1.64 | -2.05 | -0.24 |
| Siu 2006 | MACE, SBP | >7.6 | 9.84 | 1.44 | -1.86 | -0.19 |
| Szwejkowski 2013 | MACE | - | 9.08 | 2.19 | -2.15 | -0.24 |
| Taheraghdam 2014 | MACE, MACE AND PRIOR CVD | >8.2 (male)  >6.5 (female) | 8.58 | 2.22 | - | - |
| Takir 2015 | MACE | >7  (male)  >6.5 (female) | 7.67 | 0.78 | -1.04 | -0.14 |
| Tanaka 2015 | MACE | ≥7 | 7.95 | 0.99 | -1.30 | -0.16 |
| Tani 2015 | MACE | ≥7 | 7.57 | 1.03 | -1.16 | -0.15 |
| Tausche, 2017 | MACE | ≥6.5 | 9.33 | 1.51 | - | - |
| Togha 2007 | MACE | - | - | - | - | - |
| Tsuruta 2015 | MACE | ≥7 | 8.25 | 0.84 | -1.88 | -0.23 |
| Usharani 2016 | MACE | ≥6 | 8.05 | 0.95 | -1.95 | -0.24 |
| Wada 2018 | MACE | - | 7.18 | 1.17 | -2.01 | -0.28 |
| Wang 2012 | MACE | NA | - | - | - | - |
| Wang 2015 | MACE | NA | - | - | - | - |
| Weiser 2012 | MACE | - | - | - | - | - |
| Weiser 2014 | MACE | - | - | - | - | - |
| Yin 2015 | MACE, MACE AND PRIOR CVD | NA | - | - | - | - |
| Zhang 2012 | MACE, MACE AND PRIOR CVD | NA | - | - | - | - |
| Zhou 2009 | MACE | NA | - | - | - | - |
| Ziaee 2006 | MACE | - | - | - | - | - |

*- : none / not reported; NA: not available; MACE: major adverse cardiovascular events; SBP: systolic blood pressure; CVD: cardiovascular disease*

## Figure S1 – Scatter plot of the association for the instrument variants with serum urate and coronary heart disease (CHD) risk.


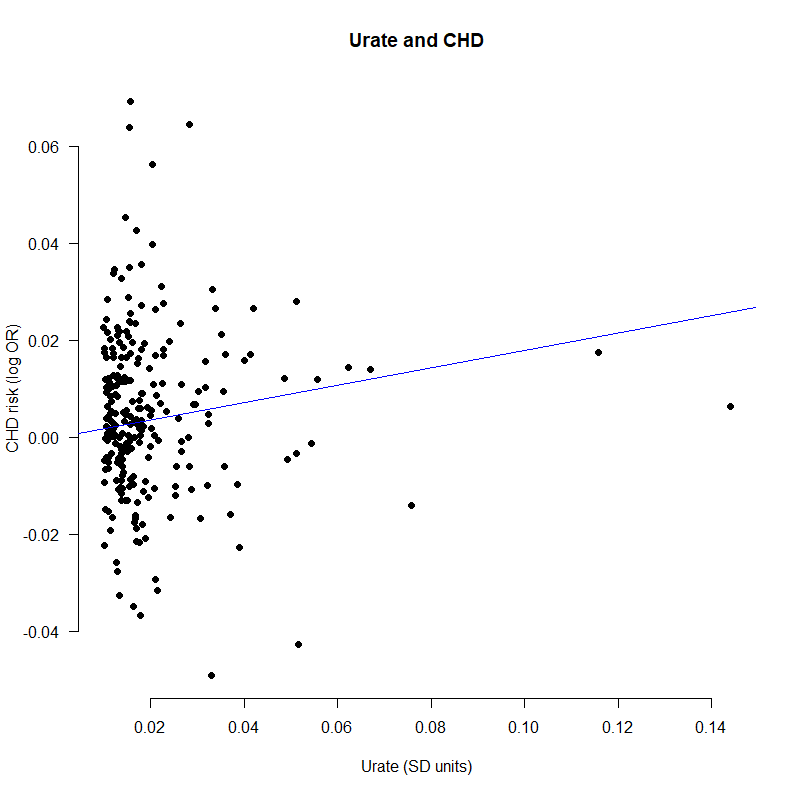


*The blue line depicts the main (inverse-variance weighted) Mendelian randomization estimate. OR: odds ratio, SD: standard deviation.*

## Figure S2 – Scatter plot of the association for the instrument variants with serum urate and peripheral arterial disease (PAD) risk.


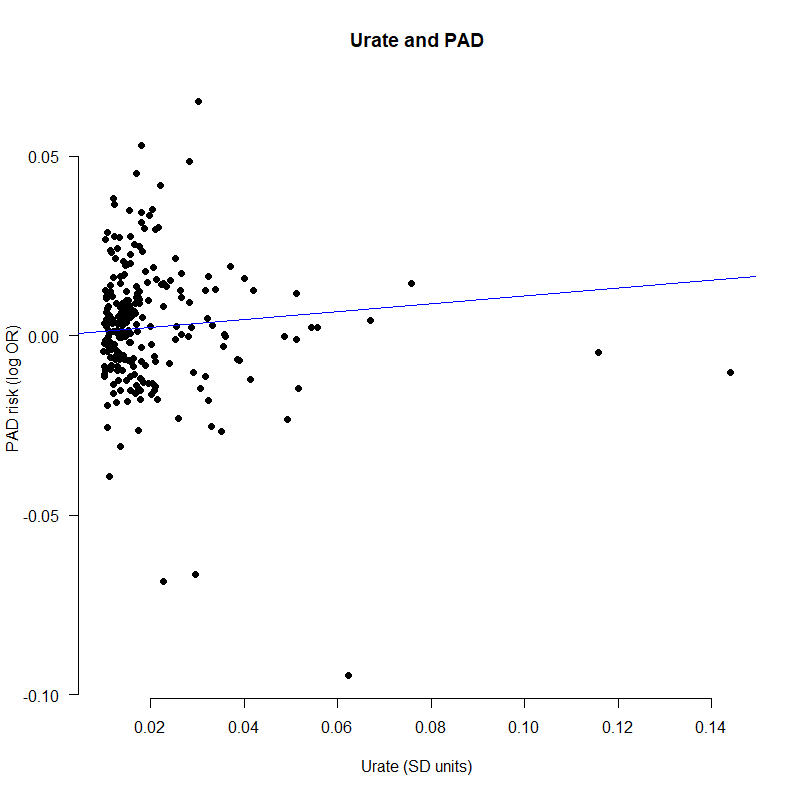


*The blue line depicts the main (inverse-variance weighted) Mendelian randomization estimate. OR: odds ratio, SD: standard deviation.*

## Figure S3 – Scatter plot of the association for the instrument variants with serum urate and stroke risk.


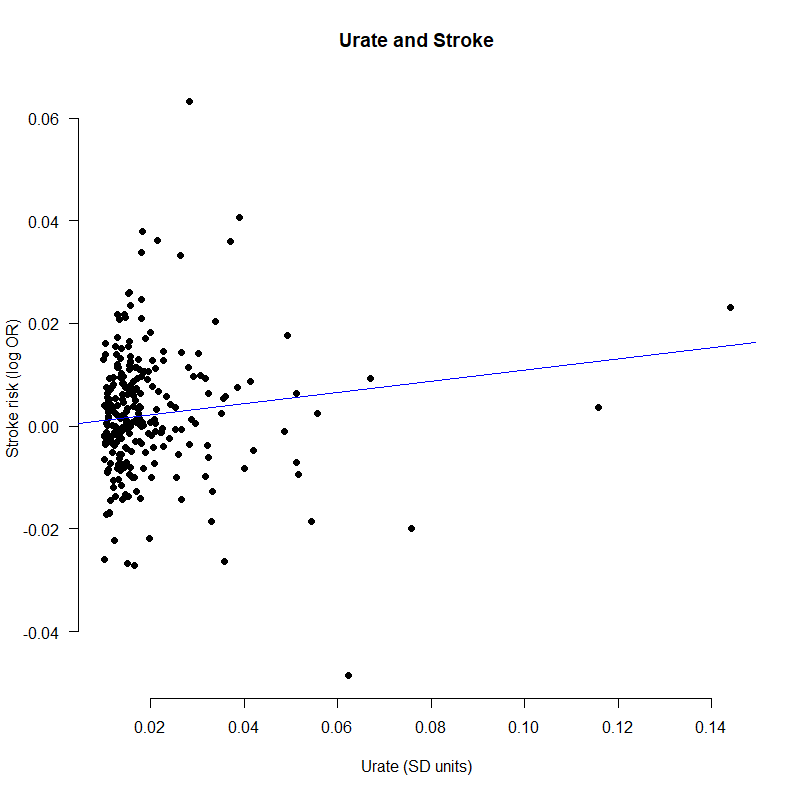


*The blue line depicts the main (inverse-variance weighted) Mendelian randomization estimate. OR: odds ratio, SD: standard deviation.*

## Figure S4 – Scatter plot of the association for the instrument variants with serum urate and systolic blood pressure (SBP).


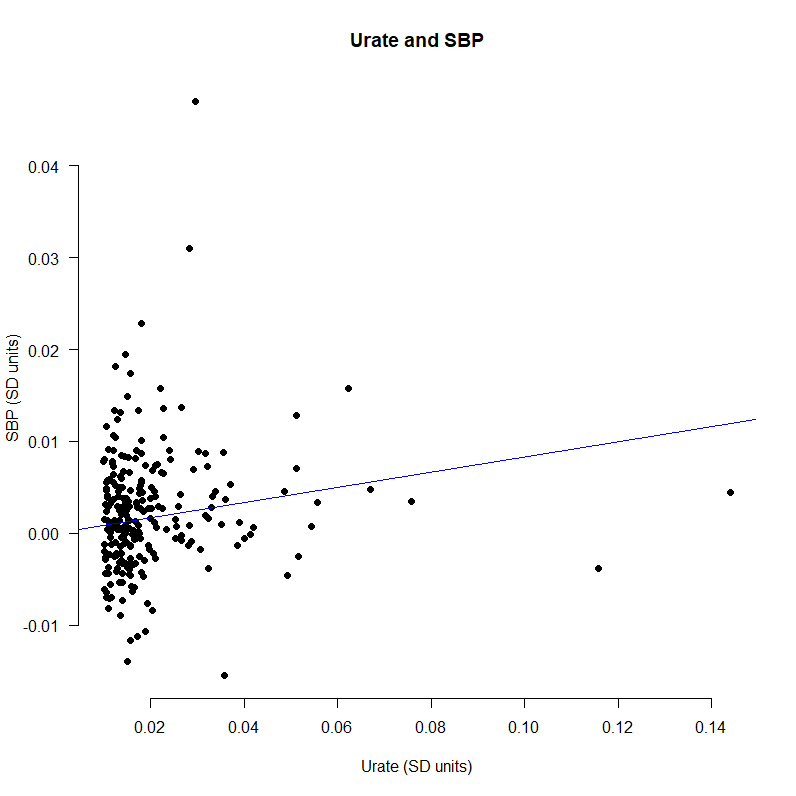
*The blue line depicts the main (inverse-variance weighted) Mendelian randomization estimate. OR: odds ratio, SD: standard deviation.*

## Figure S5 – Inverse-variance weighted (IVW) and multivariable Mendelian randomization (MVMR) estimates for the effect of 1-standard deviation (SD) increase in genetically determined serum urate levels on risk of coronary heart disease (CHD), peripheral artery disease (PAD) and stroke. The MVMR analysis adjusts for the association of the genetic instruments with systolic blood pressure, diastolic blood pressure and pulse pressure.


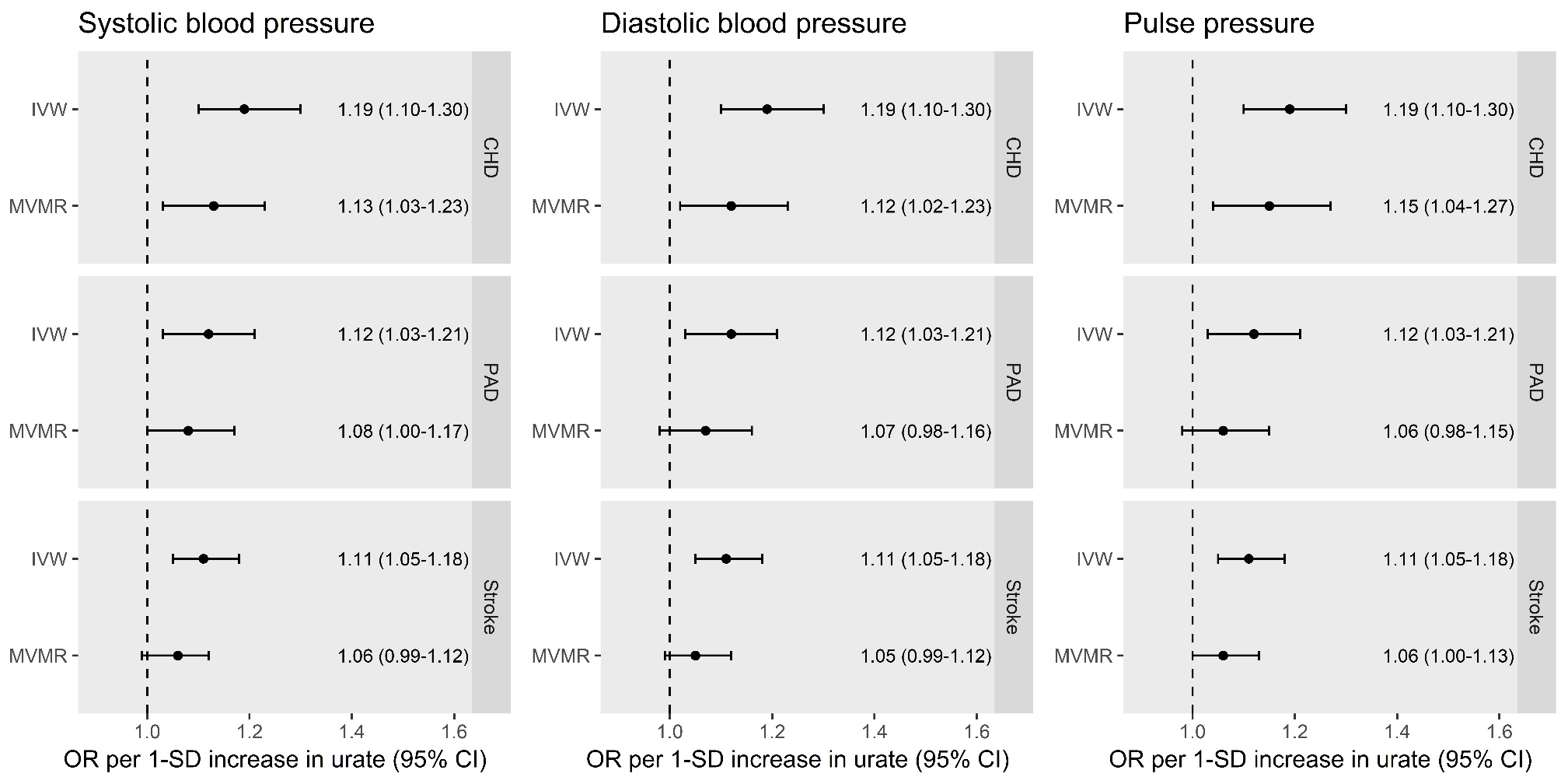


*CI: confidence interval; OR: odds ratio.*

## Figure S6 – Inverse-variance weighted (IVW) and multivariable Mendelian randomization (MVMR) estimates for the effect of 1-standard deviation (SD) increase in genetically determined serum urate levels on risk of ischaemic stroke. The MVMR analysis adjusts for the association of the genetic instruments with systolic blood pressure.


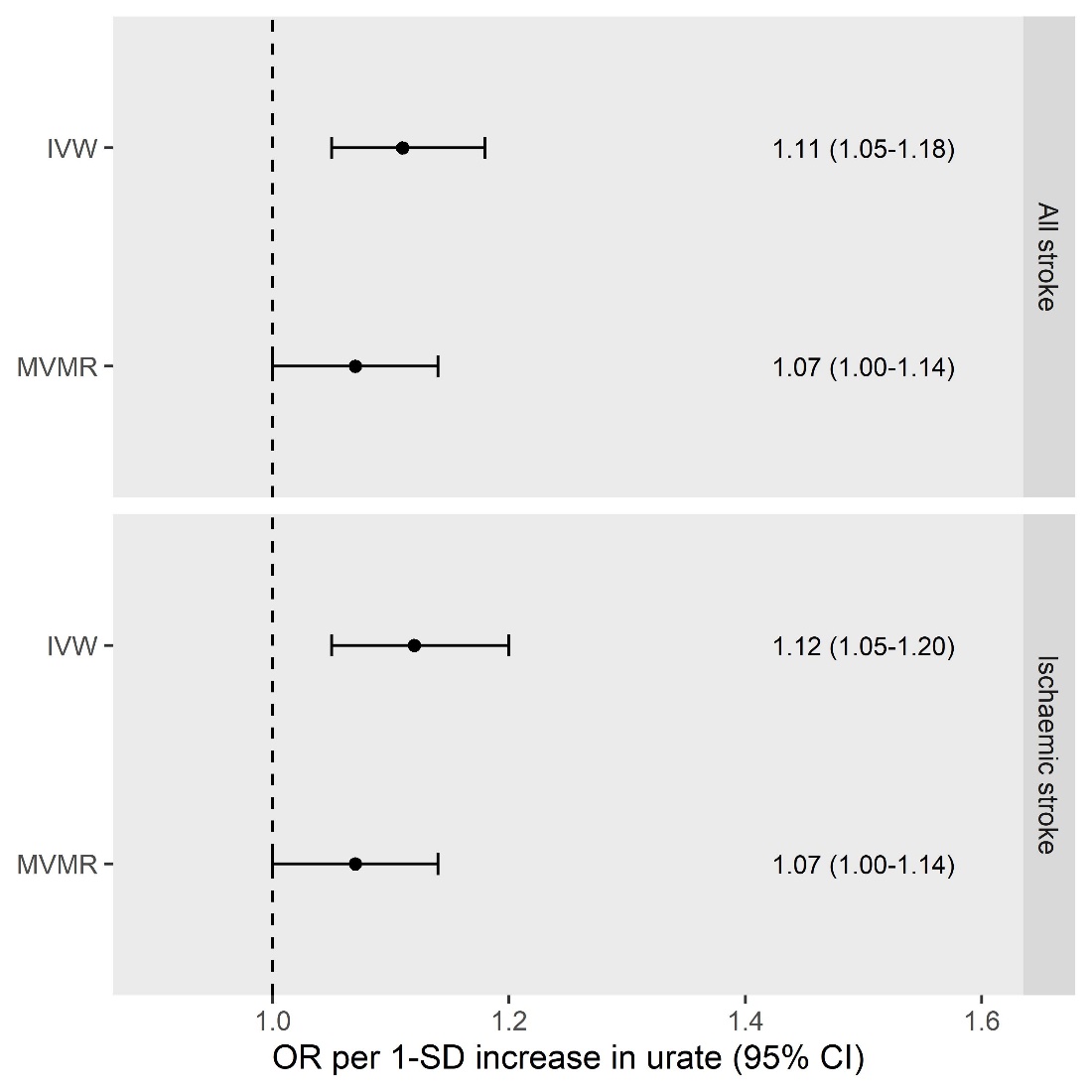


*CI: confidence interval; OR: odds ratio.*

## Figure S7 – Preferred Reporting Items for Systematic Reviews and Meta-Analyses (PRISMA) flow diagram.

**Bibliographic databases searched**:

Medline (OVID)

EMBASE (OVID)

Web of Science (Thomson Reuters)

Cochrane Library

Screening

Included

Eligibility

Identification

5353 records identified using outlined search terms

5353 records screened

Excluded based on title and abstract: 5323

30 full text articles assessed for eligibility

Excluded full text articles (17)

Already evaluated: 1

No placebo control: 2

Differing treatment groups: 1

Inadequate treatment duration: 1

Inadequate information on trial protocol: 1

Did not report outcomes of interest: 6

Did not report baseline data: 1

Non-standard urate lowering intervention: 4

No further records identified by hand search of reference lists

13 studies fulfilling eligibility criteria

## Figure S8 – Forest plot of randomized controlled trial estimates for change in mean systolic blood pressure in patients receiving urate-lowering therapy or placebo/no treatment.

*A random-effects meta-analysis model was used. I^2^ heterogeneity statistic: 89%. CI: confidence interval*

## Figure S9 – Forest plot of randomized controlled trial with low risk of bias estimates for risk of major adverse cardiovascular events in all patients receiving urate-lowering therapy or placebo/no treatment in studies.

*A random-effects meta-analysis model was used.* *I^2^ heterogeneity statistic: 0%. CI: confidence interval*

## Figure S10 – Forest plot of randomized controlled trial with low risk of bias estimates for risk of major adverse cardiovascular events in patients with existing cardiovascular disease receiving urate-lowering therapy or placebo/no treatment in studies.

*A random-effects meta-analysis model was used.* *I^2^ heterogeneity statistic: 0%. CI: confidence interval*

## Figure S11 – Meta-regression analysis investigating the association of baseline systolic blood pressure (x-axis) with mean change in systolic blood pressure (y-axis) in randomized controlled trials of patients receiving urate-lowering therapy or placebo/no treatment.

**Mean change in systolic blood pressure (mmHg)**

**Mean baseline systolic blood pressure (mmHg)**

## Figure S12 – Meta-regression analysis investigating the association of baseline serum urate concentration (x-axis) with mean change in systolic blood pressure (y-axis) in randomized controlled trials of patients receiving urate-lowering therapy or placebo/no treatment.

**Mean change in systolic blood pressure (mmHg)**

**Mean baseline serum urate concentration (mg/dL)**
